# MiR-1254 suppresses HO-1 expression through seed region-dependent silencing and non-seed interaction with TFAP2A transcript to attenuate NSCLC growth

**DOI:** 10.1371/journal.pgen.1006896

**Published:** 2017-07-27

**Authors:** Mengfan Pu, Chenggang Li, Xinming Qi, Jing Chen, Yizheng Wang, Lulu Gao, Lingling Miao, Jin Ren

**Affiliations:** 1 Center for Drug Safety Evaluation and Research, State Key Laboratory of Drug Research, Shanghai Institute of Materia Medica, Chinese Academy of Sciences, Shanghai, China; 2 School of Life Science and Technology, ShanghaiTech University, Shanghai, China; 3 University of Chinese Academy of Sciences, Beijing, China; 4 The Brain Science Center, Beijing Institute of Basic Medical Sciences, Beijing, China; University of Maryland Medical School, UNITED STATES

## Abstract

MicroRNAs (miRNAs) are a class of small non-coding RNAs, which direct post-transcriptional gene silencing (PTGS) and function in a vast range of biological events including cancer development. Most miRNAs pair to the target sites through seed region near the 5’ end, leading to mRNA cleavage and/or translation repression. Here, we demonstrated a miRNA-induced dual regulation of heme oxygenase-1 (HO-1) via seed region and non-seed region, consequently inhibited tumor growth of NSCLC. We identified miR-1254 as a negative regulator inhibiting HO-1 translation by directly targeting HO-1 3’UTR via its seed region, and suppressing HO-1 transcription via non-seed region-dependent inhibition of transcriptional factor AP-2 alpha (TFAP2A), a transcriptional activator of HO-1. MiR-1254 induced cell apoptosis and cell cycle arrest in human non-small cell lung carcinoma (NSCLC) cells by inhibiting the expression of HO-1, consequently suppressed NSCLC cell growth. Consistently with the *in vitro* studies, mouse xenograft studies validated that miR-1254 suppressed NSCLC tumor growth *in vivo*. Moreover, we found that HO-1 expression was inversely correlated with miR-1254 level in human NSCLC tumor samples and cell lines. Overall, these findings identify the dual inhibition of HO-1 by miR-1254 as a novel functional mechanism of miRNA, which results in a more effective inhibition of oncogenic mRNA, and leads to a tumor suppressive effect.

## Author summary

It is generally accepted that miRNAs bind to 3`UTR of target mRNAs and direct post-transcriptional gene silencing (PTGS) via its seed sequence. Here we report a new dual regulatory mechanism of miRNA. We described that miR-1254 repressed HO-1 at post-transcriptional level by directly targeting HO-1 3’UTR via its seed sequence and also inhibited HO-1 transcription by suppressing the transcriptional factor AP-2 alpha (TFAP2A) via its non-seed sequence. MiR-1254 induced cell apoptosis and cell cycle arrest in human non-small cell lung carcinoma (NSCLC) cells by inhibiting the expression of HO-1, consequently suppressed NSCLC cell growth. Moreover, *in vivo* mouse xenograft studies also supported the inhibitory effect of miR-1254 on NSCLC growth. These findings identify the dual regulation of miR-1254 on HO-1 as a novel functional mechanism of miRNA, which results in a more effective inhibition on the oncogenic mRNA, and leads to a suppressive effect on NSCLC growth, thus significantly advance our understanding of miRNA-directed gene regulation.

## Introduction

MicroRNAs (miRNAs) are a class of small non-coding RNAs, which direct post-transcriptional gene silencing (PTGS) and play important regulatory roles in a vast range of cellular processes including cell differentiation, proliferation, apoptosis, and migration [1–3]. Aberrant expression of miRNAs may play an important role in tumorigenesis or cancer development through dysregulation of tumor-associated genes [4–9]. It is generally accepted that miRNA binds to 3’-untranslated regions (3’-UTRs) of target mRNAs via its seed sequence (position 2–8), resulting in degradation or translational repression of the target mRNA in mammalian cells.

For a majority of miRNAs, as few as 6nt of the seed sequence matching with the target mRNA is required for functional interaction. However, besides the canonical interaction between seed region of miRNA and the 3’-UTR of target mRNA, more and more evidence show that non-canonical miRNA-target sites can be functional as well [10–13]. For example, imperfect matches of miRNA seed region with the target can be compensated by supplemental components in near-perfect sites and function in target cleavage [12, 14–16]. Studies in mouse brain shows that only 73% of the Ago-mRNA interactions can be explained by seed matches for Ago-bound miRNAs, while the rest 27% have no predicted seed matches [17, 18]. Regions outside the seed sequence may also be necessary or sufficient to direct different non-canonical regulations [11]. For example, functional “centered sites” in miRNA which have 11–12 continuous Watson–Crick pairs complementary to the target mRNA were identified by analyzing microarray data [19]. Subsequent study demonstrated that 11-mer matches of miRNA “centered sites” to the target mRNA with single mismatches or GU wobbles also form hybrids but only a small proportion leads to a repression [20], which indicates additional mechanism besides sequence complementarity may also be necessary.

The most classical function of miRNA is to induce post-transcriptional gene silencing, through either mRNA cleavage and/or translational repression. The function of miRNA has been extended to transcriptional levels, either directly or indirectly. Several studies demonstrate sequence complementarity between miRNA and target gene promoter lead to gene silencing at transcriptional level [21–27]. MiR-552 is found with a dual inhibition on CYP2E1 expression, targeting both CYP2E1 promoter via its non-seed sequence and CYP2E1 mRNA 3`UTR region via its seed sequence, respectively. It consequently induces a dual inhibition of the target mRNA at both transcriptional and post-transcriptional levels, which represents a model of effective gene regulation by miRNA [28]. Here, during our study of miRNAs regulating heme oxygenase-1 (HO-1) expression, we found another type of “dual regulation” including indirect transcriptional silencing and direct post-transcriptional inhibition.

HO-1 is a rate-limiting enzyme that metabolizes heme to generate carbon monoxide (CO), ferrous iron, and biliverdin; biliverdin is subsequently reduced to bilirubin by biliverdin reductase [29, 30]. Although the physiological HO-1 expression is only found in normal liver and spleen, HO-1 is highly induced in different types of tumors, including melanoma [31], glioblastoma [32], pancreatic cancer [33], prostate cancer [34] and non-small-cell lung cancer [35]. A growing number of studies have demonstrated that HO-1 modulated tumor growth by regulating apoptosis and cell cycle, stimulating angiogenesis, and inhibiting or terminating inflammatory response [36, 37]. Here we report that miR-1254 is a miRNA which down-regulated HO-1 via two distinct mechanisms. On the one hand, miR-1254 directly targets HO-1 via its seed sequence and represses HO-1 expression at post-transcriptional level. On the other hand, miR-1254 targets transcription factor AP-2 alpha (TFAP2A) which is a transcriptional activator of HO-1, via its non-seed sequence, and consequently represses HO-1 expression at transcriptional level. MiR-1254 induces cell apoptosis and cell cycle arrest in NSCLC cells by inhibiting the expression of HO-1, consequently suppresses NSCLC cell growth. HO-1 expression is inversely correlated with miR-1254 level in human NSCLC tumor samples and cell lines. Collectively, these findings identify the dual inhibition of HO-1 through miR-1254 as a novel functional mechanism of miRNA, which results in a more effective inhibition of oncogenic mRNA, and leads to a tumor suppressive effect.

## Results

### Bioinformatics and experimental screening identify miR-1254 as a negative regulator of HO-1

**Fig 1:**
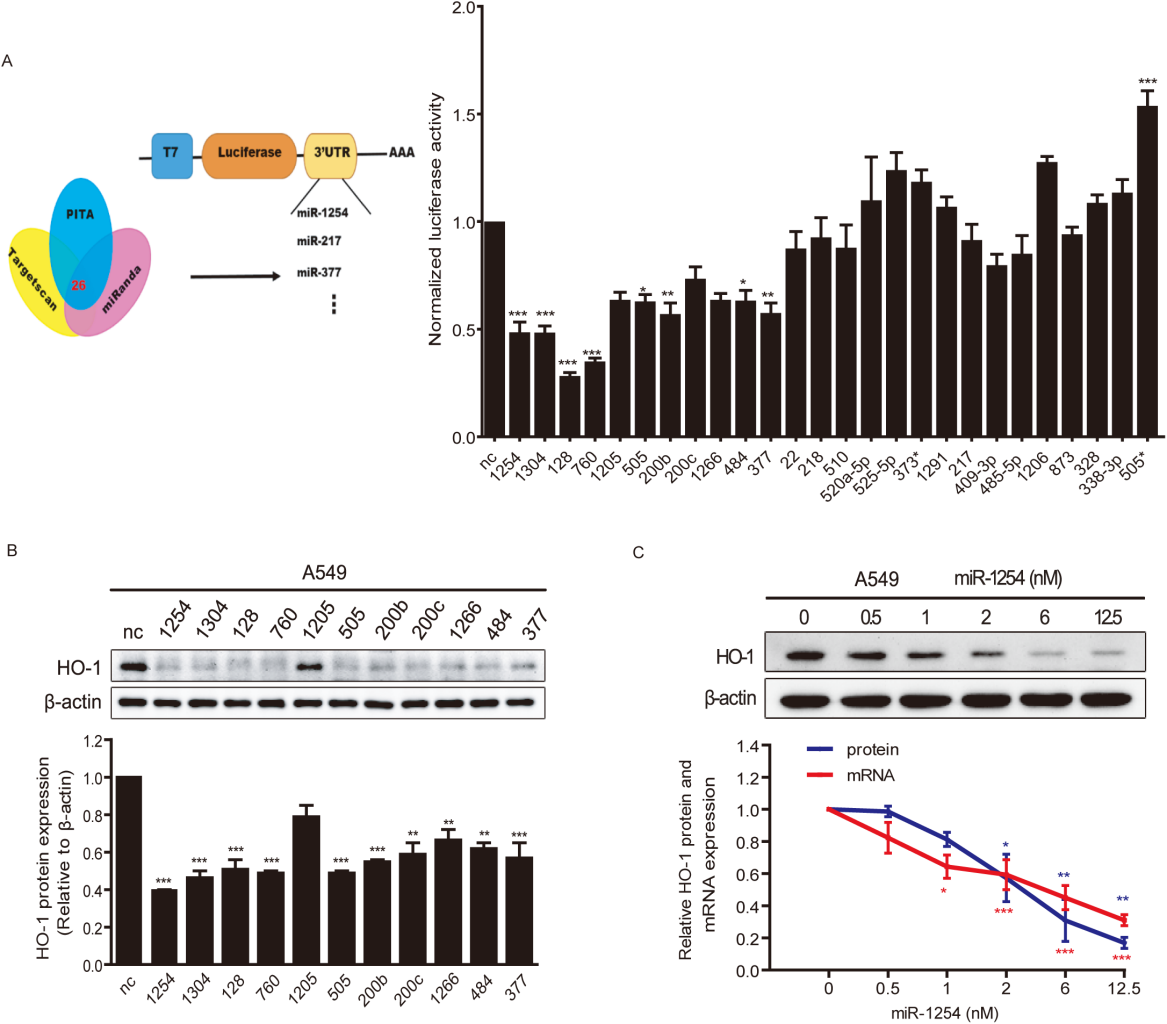
Bioinformatics and experimental screening identify miR-1254 as a negative regulator of HO-1. (A) Venn diagrams showing the number of microRNAs potentially bind to the 3’UTR of HO-1 using three predicting search programs of TargetScan, miRanda and PITA. The inhibition effects of predicted miRNAs on HO-1 3`UTR were verified by luciferase assay. (B) Upper: Immunoblots of the extracts from A549 cells transfected with indicated miRNA mimics. Bottom: Quantification of HO-1 protein levels in A549 cells normalized to β-actin. (C) HO-1 protein or mRNA levels in A549 cells transfected with the indicated doses of miR-1254 for 48h. Upper: Representative blots; nc, negative oligonucleotides. Bottom: Quantification of HO-1 mRNA and protein levels normalized to β-actin (mRNA or protein) levels and plotted as fold changes relative to the values in cells transfected with nc. Data are presented as the mean ± SEM of three independent experiments. *P<0.05, **P and ***P <0.01 vs. nc.

HO-1 over-expression has been reported to be involved in tumor growth and malignant progression [31–35], previous study from our laboratory has demonstrated that HO-1 is down-regulated by miR-1304 in lung cancer cell lines [37]. In order to find more effective miRNAs, we explored the miRNAs that potentially bind to the 3’UTR of HO-1 using bioinformatics tools. Twenty-six miRNAs were predicted to target HO-1 using all three databases (TargetScan [38], miRanda [39] and PITA [10]) (Fig 1A, left). To confirm whether these miRNAs target the 3`UTR of HO-1, we constructed a dual luciferase reporter (psi-HO1) by cloning human HO-1 3`UTR into the psiCHECK2 vector. Through co-transfection of 26 miRNAs individually with psi-HO1 reporter into HEK293 cells, we found that 11 miRNAs potently reduced the luciferase activity of psi-HO1 reporter (Fig 1A, right), indicating that these miRNAs potentially targeting HO-1 3`UTR and inhibiting HO-1 expression. To corroborate this finding, we transfected these miRNAs into human NSCLC A549 cells, western blot assays showed that miR-1254 had the strongest and most stable inhibitory effect on HO-1 protein expression (Fig 1B). We transfected different doses of miR-1254 into A549 cells, and found that miR-1254 suppressed HO-1 expression at both protein and mRNA levels in a dose-dependent manner (Fig 1C). However, the inhibition at mRNA and protein levels were not perfectly consistent, in low doses of miR-1254 (0.5~1nM), the inhibition at mRNA level is stronger than protein level, while in higher doses of miR-1254 (6~12.5nM), more dramatic inhibition was found at protein level, these results indicated that the suppression of HO-1 at mRNA and protein levels was probably achieved through different mechanisms. As a miRNA with the most dramatic inhibitory effects on HO-1 protein expression, and potentially functioning through multiple mechanisms, miR-1254 attracted our further interest.

### Both over-expressed and endogenous miR-1254 inhibit HO-1 expression in human NSCLC cells at both mRNA and protein levels

**Fig 2:**
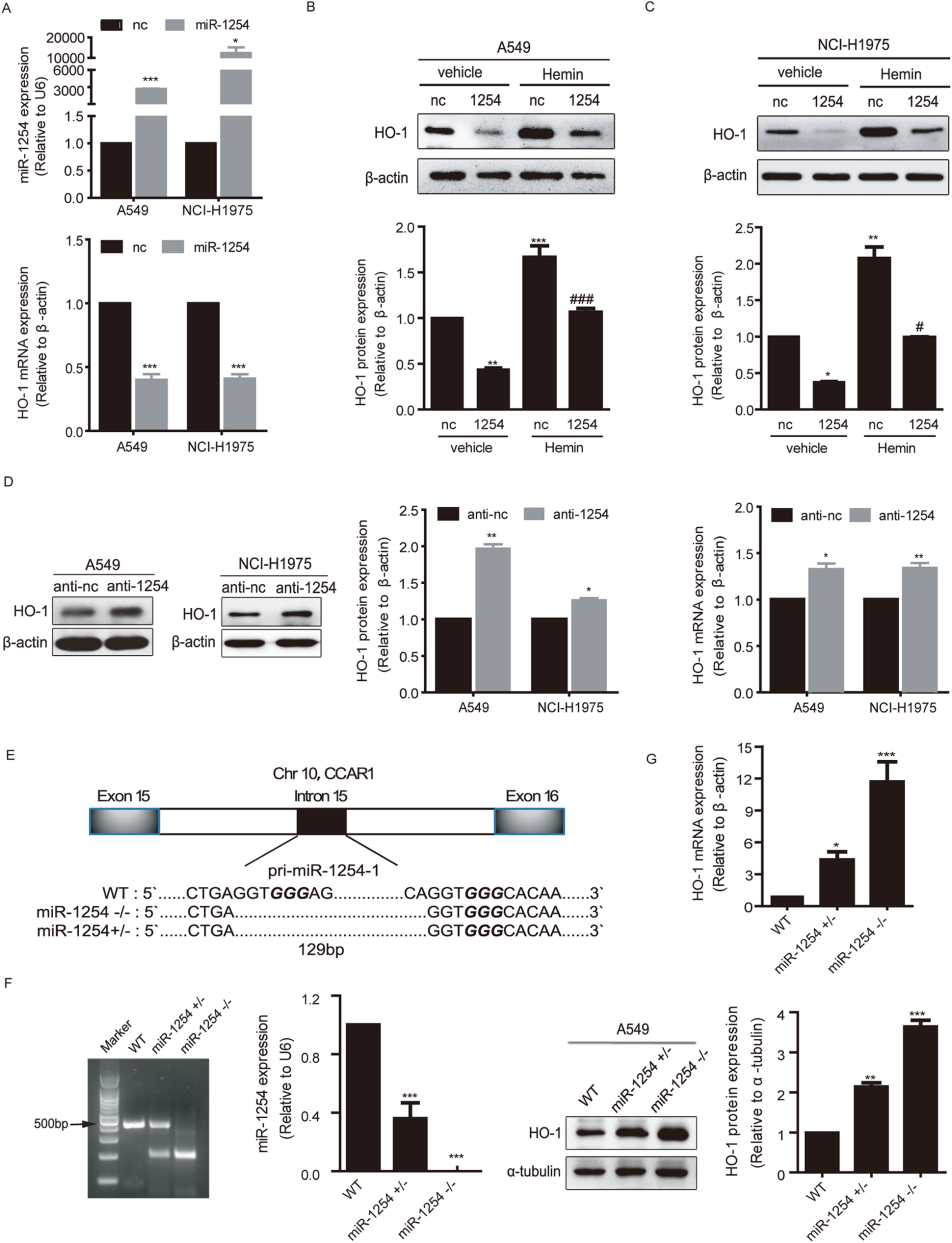
miR-1254 inhibits HO-1 expression. (A) MiR-1254 and HO-1 mRNA expression in A549 and NCI-H1975 cells transfected with miR-1254 mimics or negative control oligonucleotides (nc). (B and C) Western blot analysis of the HO-1 protein levels in the miR-1254 transfected NSCLC cell lines A549 or NCI-H1975. 20μM hemin was used to rescue the expression of HO-1 as a inducer. Upper: Representative blots. Bottom: Quantification of HO-1 protein levels normalized to β-actin protein levels and plotted as fold changes relative to the levels in cells transfected with nc. (D) HO-1 protein (left and middle) and mRNA (right) levels in NSCLC cell lines A549 or NCI-H1975 transfected with the indicated miRNA antisense oligonucleotides (Anti-1254), normalized to β-actin mRNA or protein levels and expressed relative to values in cells transfected with negative control antisense oligonucleotides (Anti-nc). Left: Representative immunoblots. 25nM of miRNA mimics or antisense oligonucleotides used for all experiments unless stated, cells harvested 48 h post-transfection. β-actin served as a loading control. Middle: Quantification of HO-1 protein levels normalized to β-actin. Right: HO-1 mRNA levels in A549 and NCI-H1975 cells. (E) Schematic representation of the CRISPR/Cas9 modified deletion of pri-miR-1254-1. The PAM sequence is italic. (F) Left: Genotyping of CRISPR/Cas9 modified miR-1254 knockdown A549 cells. Right: Taqman RT-PCR measurement of the expression level of miR-1254 in miR-1254 knockdown A549 cells. (G) HO-1 mRNA (upper) or protein (bottom) levels in CRISPR/Cas9 modified miR-1254 knockdown A549 cells. Data are presented as the mean ± SEM of three independent experiments. *P<0.05,**P and ***P <0.01 vs. nc; #*P*<0.05, ###*P*<0.01 vs. miR-1254.

We next sought to study the regulation of HO-1 expression by miR-1254 at both mRNA and protein levels in lung cancer cell lines. Human NSCLC A549 and NCI-H1975 cells were transfected with miR-1254 mimics for 48 hours, and then the expression levels of mature miR-1254, HO-1 protein and mRNA were examined. Taqman microRNA assay confirmed that miR-1254 mimics were successfully transfected into the cells and the level of mature miR-1254 was increased (Fig 2A, top). Consequently, the mRNA and protein levels of HO-1 were down-regulated in the cells transfected with miR-1254 mimics compared to that with the negative control oligonucleotides (Fig 2A bottom, 2B and 2C). We induced the expression of HO-1 in miR-1254-transfected and non-transfected cells via treatment with 20 μmol/L hemin, which is previously described as a HO-1 inducer [40]. In the presence of hemin, we found that HO-1 expression was greatly increased, and transfection of miR-1254 mimics inhibited the expression of induced HO-1 as well, to a lesser extent (Fig 2B and 2C). Taken together, our results demonstrated that miR-1254 mimics inhibited the expression of HO-1 in lung cancer cells. We subsequently explored whether the endogenous miR-1254 in NSCLC cells functions in the maintenance of HO-1 expression. MiR-1254 specific antisense oligonucleotides (Anti-1254) were used to down-regulate the level of endogenous miR-1254. Our results showed that the protein (Fig 2D, left and middle) and mRNA (Fig 2D, right) levels of HO-1 were up-regulated in A549 and NCI-H1975 lung cancer cells transfected with Anti-1254, compared to that with the negative control antisense oligonucleotides. In addition, CRISPR/Cas9 method was used to delete the genomic sequence of miR-1254 and further determine the endogenous relationship between miR-1254 and HO-1 (Fig 2E). The results showed that deletion of miR-1254 genomic sequence by CRISPR/Cas9 diminished endogenous miR-1254 level in A549 cells (Fig 2F). We established miR-1254 +/- and miR-1254 -/- cell lines derived from single clones, and examined HO-1 mRNA and protein levels in wild-type (WT), miR-1254 +/- and miR-1254 -/- cells. As expected, the expression of HO-1 at both mRNA and protein levels are negatively correlated with the level of miR-1254 in a dose-dependent manner (Fig 2G). Taken together, all these results suggest that both overexpressed and endogenous miR-1254 inhibit the expression of HO-1.

### Inhibition of HO-1 at protein but not mRNA levels were dependent on miR-1254 seed sequence through post-transcriptional gene silencing

**Fig 3:**
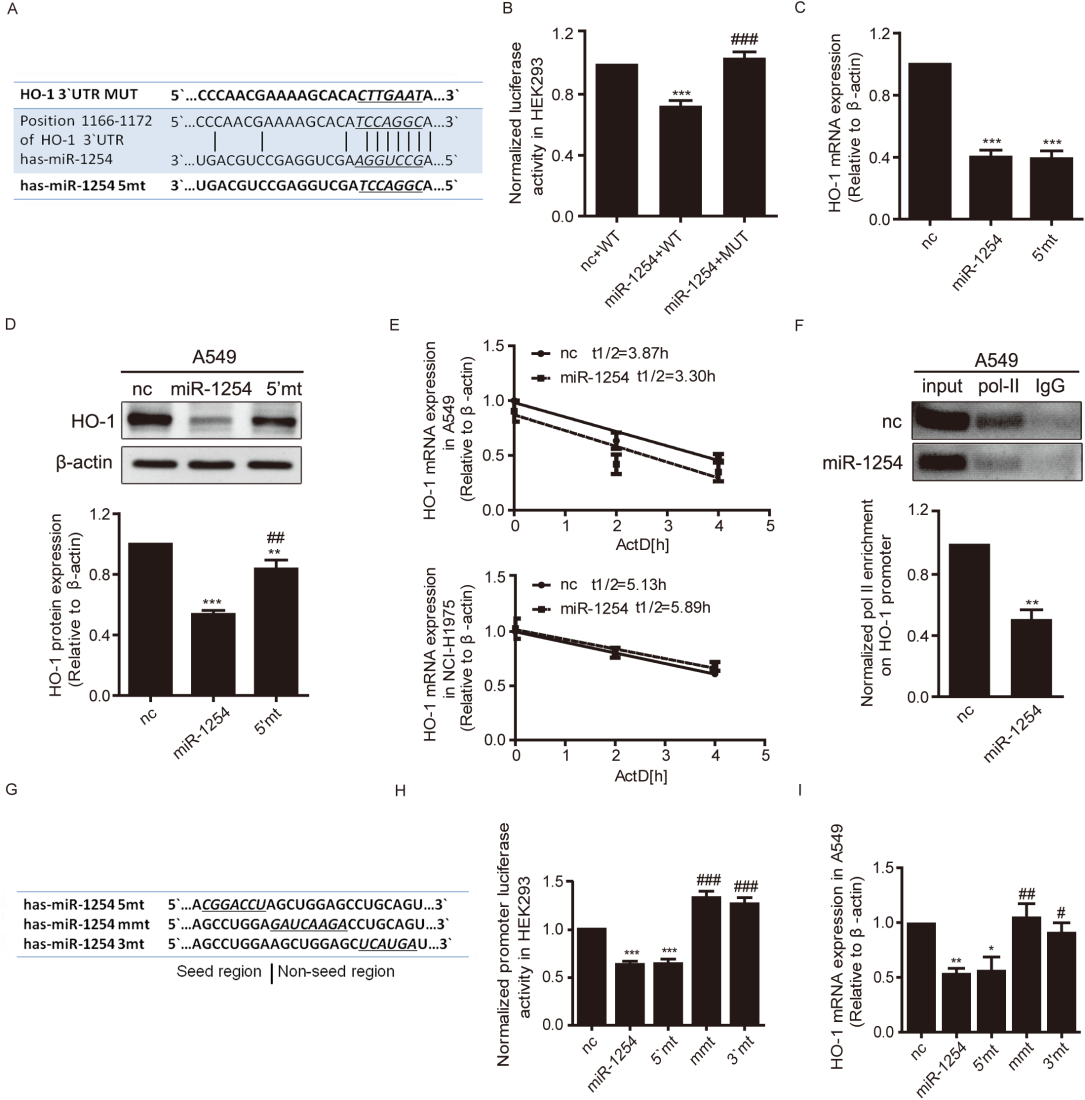
miR-1254 represses HO-1 transcription. (A) Sequence alignment of wild-type/5mt miR-1254 with wild-type/mutant HO-1 3`UTR. (B) Luciferase activity in HEK293 cells transfected with miR-1254 mimics and reporter plasmids containing wt or mt HO-1 3′-UTR normalized to activity in cells transfected with nc. (C and D) HO-1 mRNA (C) and protein (D) levels in A549 cells transfected with the indicated nucleotides (MiR-1254 and its 5`mt) for 48 h were measured by qRT-PCR and immunoblotting respectively. (E) The effect of miR-1254 on HO-1 mRNA stability. A549 and NCI-H1975 cells were transiently transfected with the indicated oligonucleotides, and after 4 hours each culture was treated with 5 μg/ml actinomycin D (ActD) for the indicated times. (F) ChIP analysis of the polⅡ binding to the HO-1 promoter in the A549 cells using antibodies against polⅡ, with IgG as a negative control. Top: Representative images. Bottom: Statistical results. (G) Nucleotides with mutations (underlined and italic) used in all experiments. (H and I) Luciferase activity in HEK293 cells (H) and HO-1 mRNA expression in A549 cells (I), cells were all transfected with indicated oligonuclitides and harvested 48h post-transfection. Data are presented as the mean ± SEM of three independent experiments. *P <0.05, **P and ***P <0.01 vs. nc; #*P* <0.05, ##*P* and ###*P* <0.01 vs miR-1254.

Since miRNAs usually direct post-transcriptional gene silencing through seed sequence binding to the 3’-UTR region of the target mRNA, we investigated the post-transcriptional effects of miR-1254 on HO-1. Predicted by TargetScan, miR-1254 seed region was complementary to the sequences from 1166–1172 in 3′UTR of HO-1 mRNA (Fig 3A). We generated dual luciferase reporter constructs containing wild type or mutant HO-1 3’UTR with mutations in miR-1254 potential binding site. Co-transfection of the wild type reporter with miR-1254 mimics resulted in a decrease of the luciferase activity in HEK293 cells (Fig 3B, lane1 and 2). As expected, the effect of miR-1254 mimics on luciferase activity was abolished in cells co-transfected with the reporter containing mutation in its binding site (Fig 3B). We further examined the mRNA and protein levels of HO-1 in A549 cells transfected with either miR-1254 or its seed sequence mutant (5`mt). Intriguingly, miR-1254-induced inhibition of HO-1 at mRNA level was not affected (Fig 3C) and the inhibition at protein level was only partly abolished (Fig 3D) upon transfection with 5`mt in A549 cells.

Given that miR-1254 reduced HO-1 mRNA level in A549 and NCI-H1975 cells (Fig 2A–2C and Fig 3C and 3D), we determined HO-1 mRNA half-life to preclude the contribution of miR-1254 on HO-1 mRNA stability. A549 and NCI-H1975 cells were transiently transfected with miR-1254 and control oligonucleotides (nc), and after 4 hours, cells were treated with 5 μg/ml actinomycin D, which is an established inhibitor of mRNA transcription [41, 42], for different times. As shown in Fig 3E, the half-life of HO-1 mRNA in A549 or NCI-H1975 cells transfected with miR-1254 was unaffected compared to the control cells, suggesting that miR-1254 did not affect HO-1 mRNA stability. This indicates that the inhibition of HO-1 at mRNA level is independent on the seed region of miR-1254, and provides further evidence to support our hypothesis that miR-1254 suppresses HO-1 expression through multiple mechanisms.

### MiR-1254 suppresses HO-1 transcription via its non-seed region

To test the hypothesis that whether miR-1254 suppresses HO-1 at the transcriptional level, first, we performed chromatin immunoprecipitation (ChIP) assays in A549 cells to examine the binding of RNA polymerase Ⅱ (pol-Ⅱ) on HO-1 promoter, and found that pol-Ⅱ but not an IgG control enrichment on HO-1 promoter fragment was reduced by miR-1254 (Fig 3F). Second, we constructed a HO-1 promoter (PGL-HO1) reporter by cloning a ∼1.5 kb human HO-1 promoter into the firefly luciferase vector PGL4.10, and we found that expressing miR-1254 greatly inhibited the luciferase activity of the reporter in HEK293 cells (Fig 3H, lane1 and 2). These findings suggest that miR-1254 represses HO-1 at transcriptional level. Moreover, mutation in miR-1254 seed region did not abolish the inhibition on the luciferase activity of the reporter, which indicated the transcriptional regulation functioned through the non-seed region (Fig 3H, lane 3). We designed different mutants of miR-1254 with mutations in non-seed region, and transfected them into HEK293 cells separately. As shown in the results, the mid region mutant mmt-6 (mmt) and the 3`region mutant 3mt-5 (3’mt) eliminated the inhibition of miR-1254 on HO-1 promoter activity (S1A Fig and Fig 3H, left, lane4 and 5). So we choose the mmt and 3’mt mutants in the following studies (Fig 3G). We examined the effect of miR-1254 non-seed region mutation (mmt and 3mt) on HO-1 mRNA in A549 (Fig 3I) and NCI-H1975 (S4A Fig, left) cells using qRT-PCR. Consistently with dual luciferase report assay, both mmt and 3mt abolished the inhibitory effects on HO-1 mRNA expression. These results suggest that miR-1254 inhibits HO-1 transcription via its non-seed sequence, while the seed region is additionally responsible for the inhibition at post-transcription level, which may consequently induces a dual inhibition of HO-1.

### MiR-1254 inhibits HO-1 transcription by targeting TFAP2A

The novelty of miR-1254 non seed region induced transcriptional gene silencing (TGS) on HO-1 inspired us to explore the exact functional mechanism of its non-seed sequence. Our laboratory has reported that miR-552 binds to CYP2E1 promoter region via its non-seed sequence and induces TGS of CYP2E1 [28]. We analyzed the sequence alignment of miR-1254 with HO-1 promoter using miRBase database [39] and RNA hybrid [43], there were 6 potential binding sites in the fragment within 1.5kb upstream from the transcription start site (TSS) (S2A Fig, top). Non-denaturing PAGE experiment was performed to test the binding ability of miR-1254 with these 6 sites in vitro, and data showed that site 2 had the highest possibility to form hybrids with miR-1254 (S2A Fig, bottom). Next we used CRISPR/Cas9 to knockout site 2 in HO-1 promoter, however, the inhibition of miR-1254 on HO-1 mRNA level was not affected (S2B and S2C Fig). These results suggest that site 2 is not the functionally targeting motif in HO-1 promoter. Subsequently, we cloned 6 fragments of HO-1 promoter with varying length (S3A Fig) into the firefly luciferase vector PGL4.10 and individually co-expressed with miR-1254 in HEK293 cells. Data showed that expressing miR-1254 greatly inhibited the luciferase activity of the reporter containing the shortest fragment 1(only 150 bp upstream from TSS) (S3B Fig). However, when we mutated both of the two potential binding sites in the fragment based on sequence alignment, the inhibition of miR-1254 on HO-1 promoter was not abolished (S3C Fig). Altogether, the results suggested that miR-1254 might not suppress HO-1 transcription via directly targeting HO-1 promoter.

Previous study showed that miRNAs can also induced DNA methylation and consequently induced transcription inhibition [44, 45]. We tested this possibility by treating A549 cells with 1μM Decitabine, a DNA demethylation drug for 48 h, after transfection with miR-1254. As shown in S3D Fig, DNA methylation was abolished, however, the inhibitory effect of miR-1254 on HO-1 transcription still existed.

**Fig 4:**
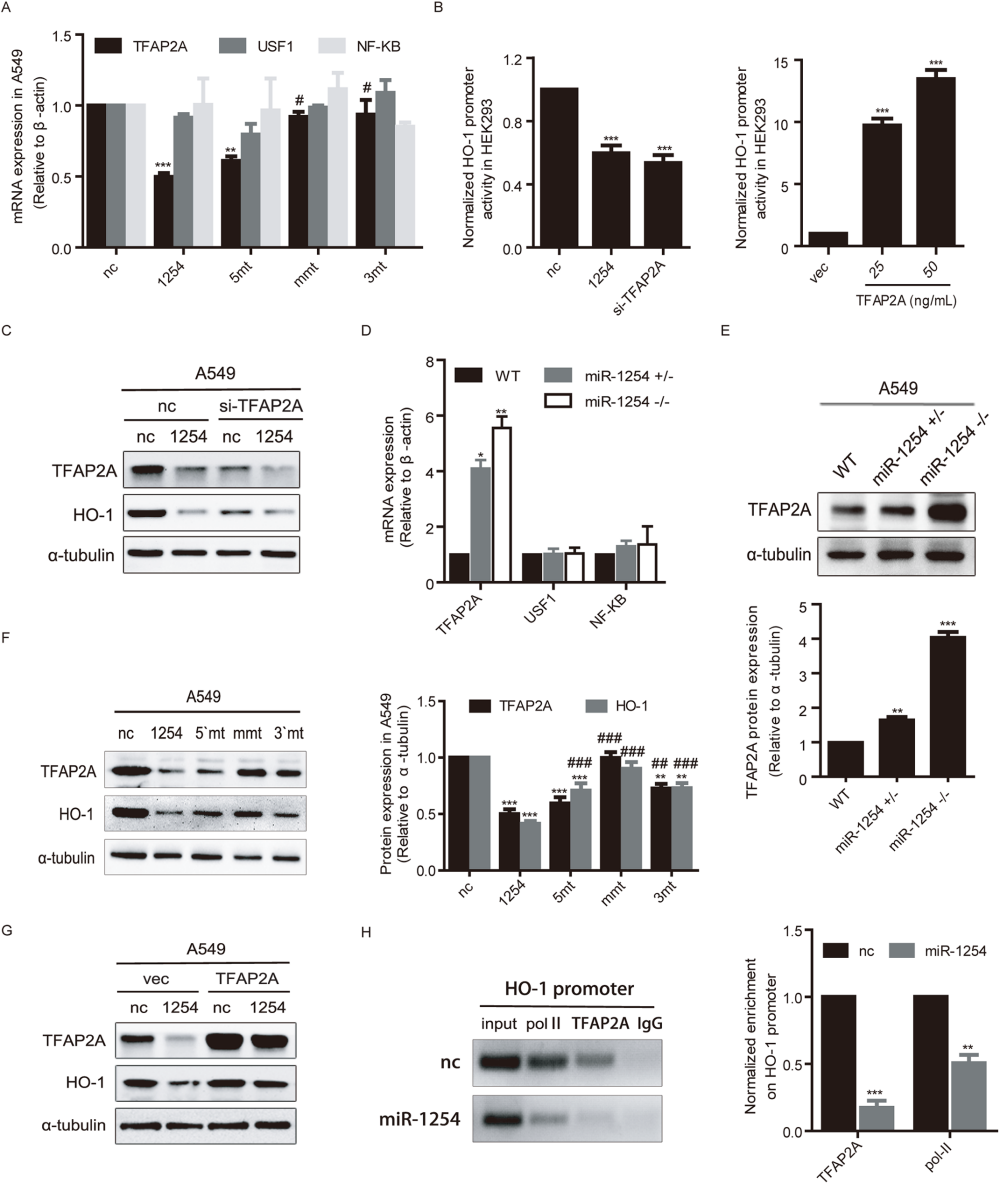
miR-1254 down-regulates HO-1 transcription via targeting TFAP2A. (A) qRT-PCR analysis of TFAP2A, USF1 and NF-κB mRNA levels in cells transfected with indicated oligonucleotides. (B) Luciferase activity assays of the PGL-HO1 reporter in HEK293 cells with TFAP2A knockdown (left) or over-expression (right). (C) Western blot analysis of the effect of TFAP2A knockdown on HO-1 protein expression compared with miR-1254 in A549 cells. (D) The mRNA levels of TFAP2A, USF1 and NF-κB in CRISPR/Cas9-modified miR-1254 knockdown A549 cells. (E) Western blot analysis of the protein level of TFAP2A in the CRISPR/Cas9-modified miR-1254 knockdown A549 cells. (F) HO-1 protein levels in cells transfected with the indicated oligonucleotides (miR-1254 and its mutants) for 48 h assayed by immunoblotting. Left: Representative images. Right: Statistical results. (G) Western blot analysis of the TFAP2A over-expression effect on HO-1 protein expression compared with miR-1254 in A549 cells. (H) ChIP analysis of the polⅡ and TFAP2A enrichment in HO-1 promoter in A549 cells using antibodies against polⅡand TFAP2A, with IgG as a negative control. Left: Representative images. Right: Statistical results. Data are presented as the mean ± SEM of three independent experiments. *P<0.05, **P and ***P <0.01 vs. nc; #*P*<0.05, ##*P* and ###*P* <0.01 vs miR-1254.

After excluding the possible mechanisms that miR-1254 directly targets HO-1 promoter or induces DNA methylation of HO-1 CpG islands we hypothesized that miR-1254 inhibits the transcription factors of HO-1 and consequently induces TGS. Plenty of regulatory elements have been identified in the promoter region of HO-1, targeted by transcriptional factors such as nuclear factor (erythroid-derived 2)-like 2 (Nrf2) [46, 47], activating protein-1 (AP-1) [48], up-stream stimulatory factor (USF) [49], nuclear factor-κB (NF-κB) and transcription factor AP-2(TFAP2) [50]. Since expressing miR-1254 inhibits the luciferase activity of the reporter containing the shortest fragment 1(only 150 bp upstream from TSS), and mutation of the binding sites on HO-1 promoter did not abolish the inhibitory effects, it’s possible that miR-1254 inhibit HO-1 transcription through targeting transcriptional factors. Predicted by TRANSFAC, TFAP2A and USF1 binding sites can be searched in this region. However, binding sites of NFκB, Nrf2 or AP-1 could not. Previous studies have demonstrated that the binding sites of NFκB are near to the binding sites of TFAP2A [46, 50], so we examined the expression of NFκB as well as TFAP2A and USF1. The results showed that TFAP2A but not USF1 or NF-κB was inhibited by miR-1254 and the inhibition effect were abolished by miR-1254 non-seed region mutants (mmt or 3mt), but not by seed region mutant (5mt), which indicated that TFAP2A may be involved in miR-1254-induced HO-1 TGS in A549 (Fig 4A) and NCI-H1975 cells (S4A Fig, right). RNA interference knockdown of TFAP2A (si-TFAP2A) potently reduced the HO-1 promoter luciferase reporter activity (Fig 4B, left). To further study the role of TFAP2A in miR-1254 regulation of HO-1, we cloned the coding sequence of TFAP2A into pTT5 vector (pTT5-TFAP2A) and then co-transfected it with PGL-HO1 into HEK293 cells. As shown in the data, the PGL-HO1 luciferase activity was substantially increased by the co-transfection with pTT5-TFAP2A in a dose-dependent manner (Fig 4B, right). We confirmed these results in A549 (Fig 4C) and NCI-H1975 cells (S4B Fig) with TFAP2A knocked down using chemically synthesized siRNA against TFAP2A (si-TFAP2A), the results consistently showed that HO-1 protein expression was decreased by si-TFAP2A. These results suggest that TFAP2A is a major transcription factor that functions in HO-1 activation in these cells.

Then, we examined the changes at mRNA and protein levels in A549 cells with CRIPSR/Cas9-modified miR-1254 knockdown. The results showed that the mRNA (Fig 4D) and protein (Fig 4E) expression levels of TFAF2A were dramatically increased in a dose-dependent manner in miR-1254-knockdown cells, but not the other two transcription factors of USF1 and NF-κB (Fig 4D), consistently with the changes of HO-1 expression at mRNA level (Fig 2G). These results suggested that the endogenous miR-1254 inhibited TFAP2A expression at both mRNA and protein levels. We then performed western blot assay to examine the effects of miR-1254 and its mutants on protein levels of TFAP2A and HO-1 in NSCLC cells. Consistently, the protein levels of both TFAP2A and HO-1 were inhibited by miR-1254 in both A549 (Fig 4F, lane1 and 2) and NCI-H1975 (S4C Fig, lane1 and 2) cells. MiR-1254 mmt basically lost the inhibition on TFAF2A and HO-1 protein expression, while miR-1254 5mt maintained the inhibitory function on TFAP2A, and partially attenuated the protein reduction of HO-1 (Figs 4F and S4C).

To confirm whether miR-1254 inhibits HO-1 mRNA expression through down-regulating TFAF2A, we co-transfected miR-1254 mimics with pTT5-TFAP2A into A549 (Fig 4G) and NCI-H1975 cells (S4D Fig), the results showed that over-expression of TFAP2A rescued miR-1254-induced inhibition on HO-1 expression. We further performed chromatin immunoprecipitation (ChIP) assays in A549 cells, and found that the enrichment of TFAP2A but not an IgG control on HO-1 promoter fragment was reduced by miR-1254, consistently with the enrichment results of polymerase Ⅱ (pol-Ⅱ) (Fig 4H). Collectively, these findings support that TFAP2A is a transcriptional activator for HO-1 in NSCLC cells and miR-1254 represses HO-1 transcription through targeting TFAP2A.

### MiR-1254 targets TFAP2A 3`UTR via its non-seed sequence

**Fig 5:**
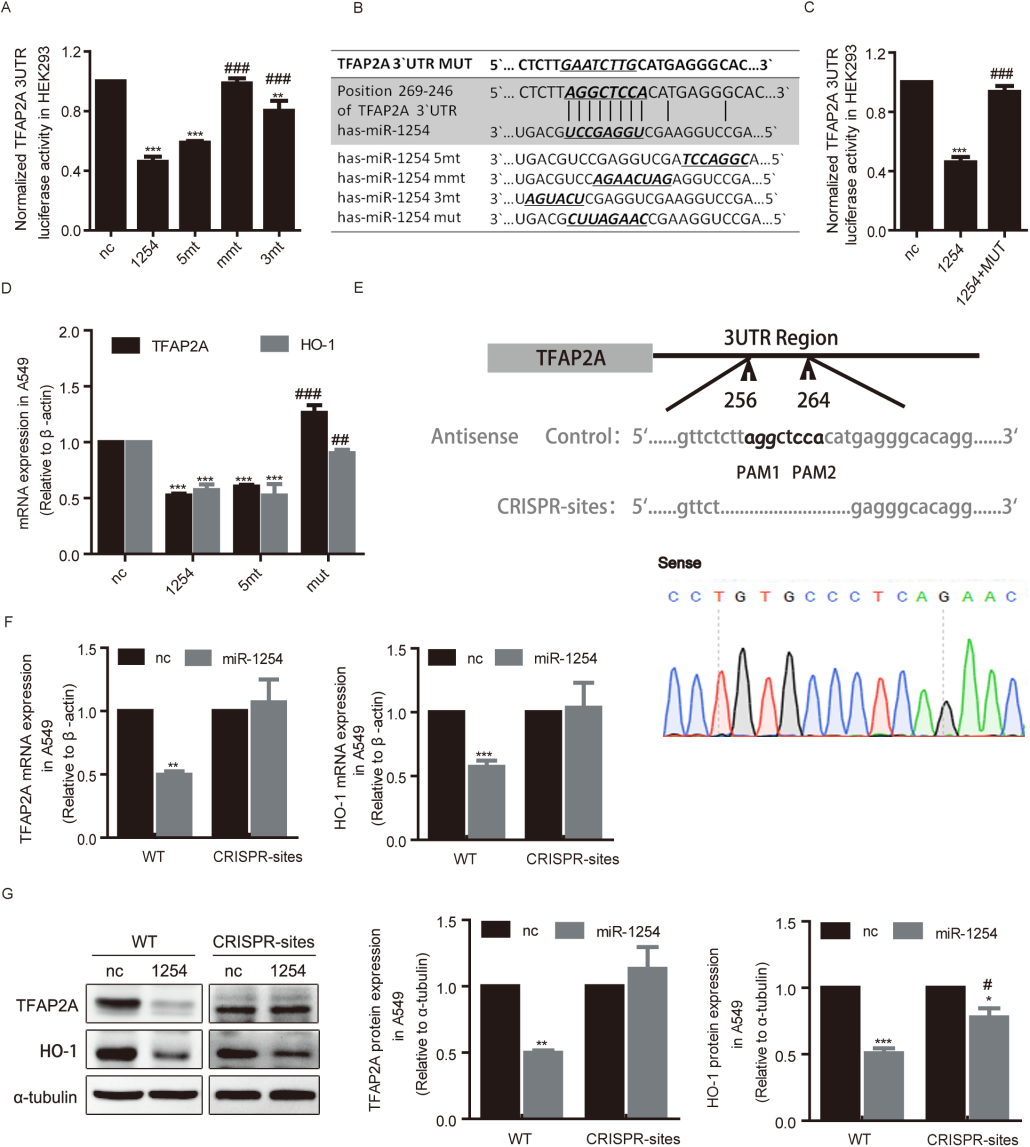
miR-1254 targets TFAP2A 3`UTR via its non-seed sequence. (A) Luciferase activity of psi-TFAP2A reporter in HEK293 cells transfected with indicated oligonucleotides. (B) Sequence complementarity between the 3′UTR of TFAP2A mRNA and the different region of miR-1254. (C) Luciferase activity in HEK293 cells transfected with miR-1254 mimics and reporter plasmids containing wt or mt TFAP2A 3′-UTR normalized to that in cells transfected with negative control (nc). (D) TFAP2A and HO-1 mRNA levels in A549 cells transfected with indicated oligonucleotides. (E) Upper: Schematic representation of the CRISPR/Cas9 modified nucleotides knockout in TFAP2A 3`UTR, the PAM sequence is italic. Bottom: DNA sequencing used to confirm the CRISPR/Cas9 modified genome editing.(F) qRT-PCR analysis of the mRNA levels of TFAP2A and HO-1 expression in the CRISPR-sites A549 cells. CRISPR-sites A549 cells: CRISPR/Cas9-modified A549 cells which the binding sites of miR-1254 on TFAP2A 3`UTR were deleted.(G) Western blot analysis of the effects of miR-1254 on TFAP2A and HO-1 expression in wild-type or CRISPR-sites A549 cell lines. Left: Representative images. Right: Statistical results. Data are presented as the mean ± SEM of three independent experiments. **P and ***P <0.01 vs. nc; #*P*<0.05,##*P* and ###*P* <0.01 vs. miR-1254.

Our results suggested miR-1254 directs TGS of HO-1 expression via targeting the transcriptional activator TFAP2A, possibly through its non-seed region. We then elucidated the regulatory mechanism through which miR-1254 suppressed TFAP2A expression. We cloned 3`UTR of TFAP2A into the luciferase reporter psi-CHECK2 (psi-TFAP2A) and co-transfected with miR-1254 mimics or its mutants with mutation in different regions into HEK293cells. Consistent with the results on HO-1 mRNA expression, the luciferase activity of psi-TFAF2A was inhibited by miR-1254, and the inhibitory effect was abolished by miR-1254 non-seed region mutants (mmt or 3mt) but not seed region mutant (5mt) (Fig 5A). Then, we analyzed the sequence alignment of miR-1254 with TFAP2A 3`UTR. Predicted by RNA hybrid, miR-1254 non-seed region was complementary to the sequence in 3′UTR of TFAP2A mRNA (from 264–257) (Fig 5B). When mutations were introduced into the 8 nt sequence in TFAP2A mRNA 3’-UTR complementary to non-seed sequence of miR-1254, miR-1254 could not suppress the activity of the mutant reporter any longer (Fig 5C). Moreover, mutations were also introduced in seed and non-seed region of miR-1254 (5’mt, mut), qRT- PCR was performed to test their effects on HO-1 in A549 cells. As shown in the results, mRNA expression of TFAP2A was rescued when miR-1254 non-seed region were mutant (Fig 5D).

CRISPR/Cas9 was used to knockout the endogenous binding site of miR-1254 on TFAP2A 3`UTR genomic sequence (Fig 5E). The results showed that the inhibitory effect of miR-1254 on TFAP2A (Fig 5F, left) and HO-1 (Fig 5F, right) mRNA level were completely abolished in CRISPR/Cas9-modified A549 cells (CRISPR-sites). In addition, western blot showed that miR-1254 could not suppress TFAP2A protein expression any longer, however, miR-1254 still strongly inhibited HO-1 protein expression in CRISPR-sites cells (Fig 5G). These results suggest that miR-1254 binds to TFAP2A 3`UTR via its non-seed sequence through an 8 nt-contiguous Watson–Crick pairs and miR-1254 represses HO-1 expression at post-transcriptional level by directly targeting HO-1 3’UTR via its seed sequence. Altogether, we found that miR-1254 suppresses HO-1 expression at mRNA and protein levels through different mechanisms, and dependent on different regions.

### MiR-1254 suppresses cell growth of NSCLC cells through HO-1 by inducing cell cycle arrest and cell apoptosis

**Fig 6:**
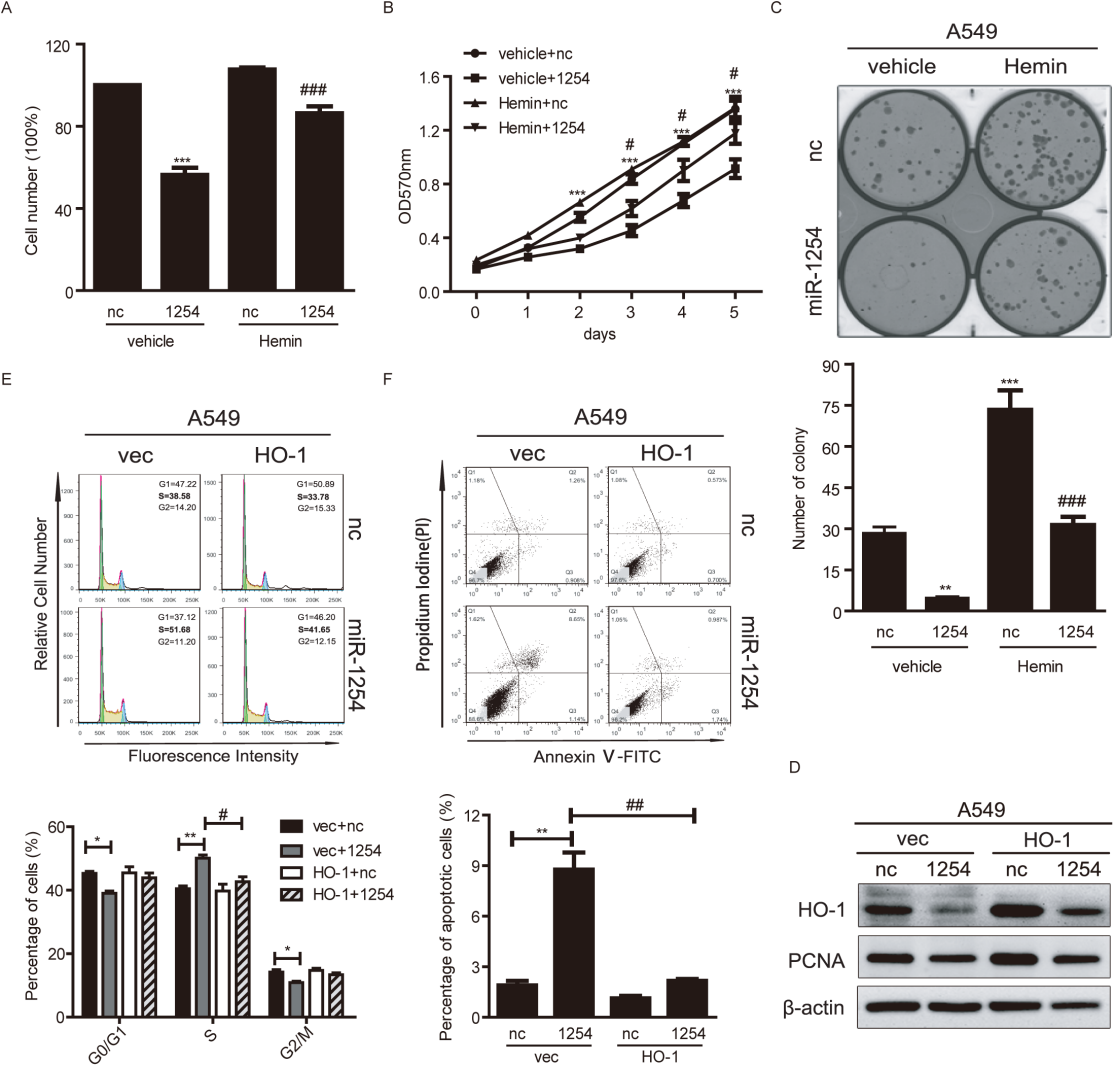
HO-1 plays a critical role in miR-1254-modified cell growth suppression of NSCLC cells. (A-C) Inhibition of cell growth by miR-1254 in A549 cells. Cells were transfected with miR-1254 mimics or negative control oligonucleotides (nc), 20μM hemin was used to rescue the expression of HO-1 as a inducer. (A) Trypan blue staining assays. Cells were counted 72h after transfection. (B) MTT analysis of cell viability in A549 cells transfected with miR-1254 mimics or nc. (C) Colony formation in A549 cells transfected with miR-1254 mimics compared with nc. Upper: Representative image of the colony formation. Bottom: Statistical results. (D) Western blot analysis of the protein levels of proliferating cell nuclear antigen (PCNA) and HO-1 in the cells transfected with indicated plasmids and oligonucleotides, β-actin served as internal normalized reference. (E and F) Flow cytometry analysis of cell cycle (E) and apoptosis (F) in A549 cells. Upper: Representative images. Bottom: Statistical results. Data are presented as the mean ± SEM of three independent experiments. *P<0.05, **P and ***P <0.01 vs. nc; #*P*<0.05, ##*P* and ###*P* <0.01 vs miR-1254.

As described previously, it has been found that HO-1 plays a vital role in promoting cell survival in several types of cancer [31–35]. It is highly possible that miR-1254 regulates human lung cancer cell growth through modulating the expression of HO-1. In order to investigate the effects of miR-1254 on lung cancer cell growth, miR-1254 mimics were transfected into A549 and NCI-H1975 cells. Trypan blue staining showed that miR-1254 over-expression for 3 days markedly decreased the number of A549 (Fig 6A) and NCI-H1975 cells (S5A Fig). MTT assay was used to examine the effects of miR-1254 on cell viability. Our results showed that the viability of A549 and NCI-H1975 cells transfected with miR-1254 mimics was clearly decreased compared to those transfected with negative control oligonucleotides (Figs 6B and S5B). In the colony formation assay, transfection with miR-1254 mimics inhibited the colony-forming activity of both A549 and NCI-H1975 cells, while transfection with negative control oligonucleotides has no such effects (Figs 6C and S5C). To determine the relationship between HO-1, miR-1254 and cell survival, a combination study was carried out whereby cells were first transfected with miR-1254 mimics, followed by treatment with 20μM hemin chloride [40]. Trypan blue staining, MTT assay and colony formation assay revealed that the decrease of cell viability due to miR-1254 transfection could be rescued by inducing HO-1 expression with hemin chloride in A549 (Fig 6A–6C) and NCI-H1975 (S5A–S5C Fig) cells. These data demonstrated that miR-1254 suppresses the growth of NSCLC cells by repressing the expression of HO-1.

To explore the precise path by which miR-1254 reduced the NSCLC cell number, we cloned the coding sequence of HO-1 into pTT5 vector (pTT5-HO1). The plasmids were transfected into NSCLC cells to overexpress HO-1 and test the rescue effects on cell proliferation, cell cycle and apoptosis. Western blot analysis of HO-1 and proliferating cell nuclear antigen (PCNA) indicated that miR-1254 over-expression in A549 cells reduced cell proliferation, as expected, the restoration of HO-1 expression strongly overrode the repression effects (Fig 6D). Flow cytometry combining with PI staining and Annexin V-FITC/PI staining assay were used to analyze the cell cycle and apoptosis, respectively. The results showed that enforced miR-1254 expression led to more than 10% S phase cell cycle arrest and a significantly higher percentage of apoptotic cells. Consistently, HO-1 re-expression attenuated miR-1254-induced S phase cell cycle arrest and cell apoptosis in A549 (Fig 6E and 6F) and NCI-H1975 (S5D Fig and S5E Fig). Altogether, these results implied that miR-1254 suppress the growth of NSCLC cells by inducing cell cycle arrest and cell apoptosis, and with a mechanism of inhibiting HO-1 expression.

### MiR-1254 suppresses NSCLC tumor growth in vivo

**Fig 7:**
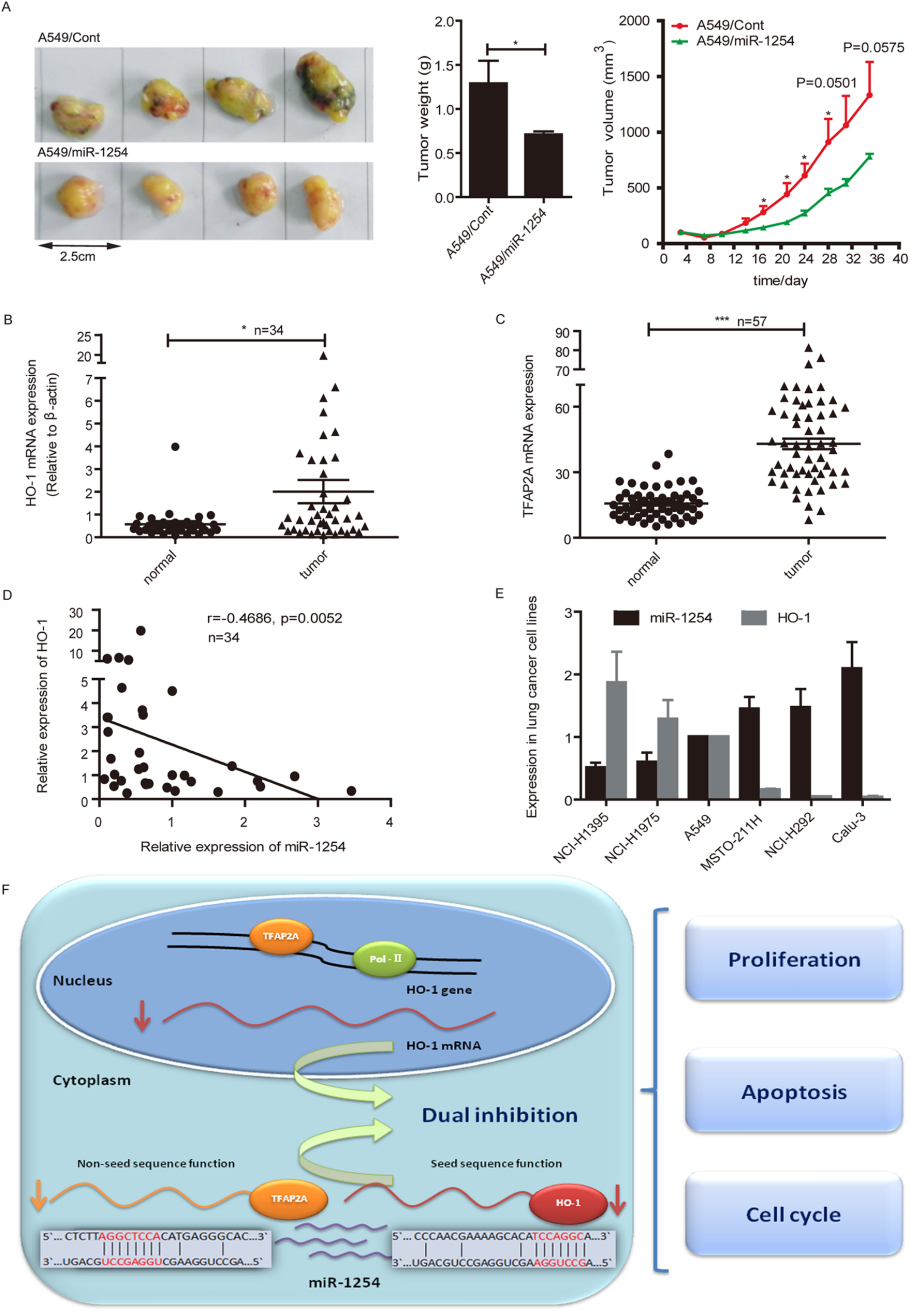
MiR-1254 suppresses NSCLC tumor growth in vivo. (A) Representative photographs of the tumors at day 35 after inoculation with either the A549/miR-1254 or A549/miR-Control cells. (B) Comparison of HO-1 mRNA levels in paired NSCLC tumor samples and normal lung tissue specimens (n = 34). (C) Analysis of the TFAP2A expression in paired tumor and normal samples from patients with NSCLC in The Cancer Genome Atlas (TCGA) database (n = 57). (D) Inverse correlation between HO-1 expression and miR-1254 level in NSCLC tumor samples. miR-1254 and HO-1 mRNA levels in tumor specimens (n = 34) were determined by qRT–PCR, with U6 andβ-actin as respective internal normalized references. The correlation between the miR-1254 and HO-1 expression in these tumors was examined using the Spearman’s correlation test. (E) qRT–PCR analysis of the expression of miR-1254 and HO-1 in the lung cancer cell lines (NCI-1395, NCI-H1975, A549, MSTO-211H, NCI-H292, Calu-3). U6 and β-actin served as internal normalized references for miR-1254 and HO-1, respectively. (F) Diagram of working model. Data in all experiments were mean ± SEM of three independent experiments in triplicates. *P < 0.05, ***P < 0.01 vs. nc.

MiR-1254 has been reported to be down-regulated in breast cancer cells. Over-expressing of miR-1254 could inhibit breast tumor growth and overrides tamoxifen resistance [51]. Our *in vitro* results suggested miR-1254 suppressed NSCLC cell growth, we also study the *in vivo* effects using mouse xenograft model. We established A549 cell line stably over-expressing miR-1254 (A549/miR-1254) by lentiviral transduction. Western blot showed that TFAP2A and HO-1 protein levels in A549/miR-1254 cells were markedly decreased compared with the cells over-expressing negative control oligonucleotides (A549/Cont cells) (S5F Fig). Then, A549/miR-1254 and A549/Cont cells were subcutaneously injected into nude mice respectively. We found that over-expression of miR-1254 in A549 cells significantly reduced tumor growth in nude mice compared with control cells (Fig 7A). These results additionally support our original finding that miR-1254 has inhibitory effects on NSCLC growth.

### HO-1 expression is inversely correlated with miR-1254 expression in human NSCLC tumor samples and cell lines

We examined the HO-1 mRNA level in 34 paired frozen NSCLC tumor samples and normal lung tissue specimens. Using quantitative reverse transcription–PCR (qRT–PCR), we found that the expression levels of HO-1 in tumor were significantly higher than those in normal lung tissues (Fig 7B). We analyzed the expression of TFAP2A in 57 paired tumor and normal samples from patients with NSCLC in The Cancer Genome Atlas (TCGA) database, and found that TFAP2A was significantly induced in tumor samples, consistently with HO-1 (Fig 7C). To characterize whether miR-1254 is involved in HO-1 regulation in human NSCLC, qRT–PCR was used to examine the levels of both miR-1254 and HO-1 mRNA in the same set of human NSCLC specimens. We found an inverse correlation between the level of miR-1254 and HO-1 mRNA expression in these tumors (Spearman’s R = − 0.4686, P = 0.0052<0.01 = (Fig 7D). The negative correlation between HO-1 and miR-1254 was also observed in multiple human lung cancer cell lines (Fig 7E). The results indicate that miR-1254 may be a negative regulator of HO-1 in human NSCLC patient samples and cell lines.

## Discussion

In general, miRNAs bind to 3`UTR of target mRNAs and direct PTGS via its seed sequence, however, we and other groups demonstrated that miRNAs can also function through its non-seed sequence [19, 28]. For example, 11–12 nt Watson–Crick paring between the center of the miRNA and the “centered sites” in target was proved to be functional in target suppression. [19, 20]. Our results suggest that miR-1254 binds to TFAP2A 3`UTR via its non-seed sequence through 8 contiguous Watson–Crick pairs effectively inhibits TFAF2A and its target gene HO- 1 expression. When we screened the miRNAs which potentially target HO-1 3’-UTR, which indicated the effect of post-transcriptional gene regulation, miR-1254 was not the one which has the most dramatic effect on HO-1 3’-UTR luciferase reporter, however, miR-1254 is the one which suppresses HO-1 protein expression most effectively, which also indicates a transcriptional inhibition in addition to the post-transcriptional gene silencing. Our data showed that HO-1 expression was negatively regulated by miR-1254 at transcriptional and post-transcriptional levels via its non-seed sequence and seed sequence, with indirect and direct mechanisms, respectively. The dual inhibition of miR-1254 we found here may represent a novel regulatory mechanism of miRNA, which results in a stronger and more stable suppression on target gene expression.

Previous studies have reported that miR-1254 expression is dysregulated in human breast cancer[51], retinoblastoma[52] and NSCLC[53, 54]. MiR-1254 was identified as a circulating miRNA downregulated in NSCLC patient serum, compared with healthy control [54]. However, in another study, miR-1254 was detected upregulated in early-stage NSCLC tumor samples, and is considered as a candidate for serum-based biomarker [53]. Those studies suggested that miR-1254 might be involved in tumor progression, but the exact function is largely unknown. As miR-1254 has a very high GC content (62.5%), it usually gives low reads in RNA-seq data, which makes it more difficult to be investigated [51].

HO-1 expression is widely up-regulated in various types of tumors and consequently impacts tumor development by promoting cancer cell growth, invasion and metastasis [55]. Previous studies showed that HO-1 can be regulated at both transcriptional and post-transcriptional levels [37, 56]. Several miRNAs have been reported as regulators of HO-1[37, 40, 57], and seven unreported miRNAs with inhibitory activity on HO-1 were screened out in our current work as shown in Fig 1B. These miRNAs are inactivated in different types of cancer cells under most circumstances. In the present study, we demonstrated that HO-1 is regulated by miR-1254 at both mRNA and protein levels in human lung cancer cells. MiR-1254 directly targets HO-1 3’-UTR via its seed sequence and represses HO-1 expression at post-transcriptional level. In parallel, miR-1254 suppresses TFAP2A, which is a transcriptional activator of HO-1, via its non-seed sequence, and consequently represses HO-1 expression at transcriptional level. This dual regulatory mechanism by miR-1254 at both transcriptional and post-transcriptional levels has the potential to lead a more effective inhibition effect on HO-1. Moreover, there are many other oncogenes which are similarly regulated with HO-1, they could be regulated both by miR-1254 and TFAP2A directly. ChIPBase was used to predict the transcriptional targets of TFAP2A and TargetScan was used to predict miR-1254 targets. The intersection of the two populations has 347 targets in total including HO-1. Importantly, our findings indicate that miR-1254 induces cell apoptosis and cell cycle arrest of NSCLC cells through inhibiting the expression of HO-1, consequently suppressing the NSCLC cell growth. The clinical data showing that HO-1 mRNA level is inversely correlated with miR-1254 in human NSCLC tumor samples, indicating that miR-1254 may be a negative regulator of HO-1 in physiological conditions. Collectively, our findings identify miR-1254 as an inhibitor of HO-1 with dual regulatory mechanisms via different sequence regions, and functioning in NSCLC cell growth inhibition (Fig 7F).

Despite the new functional mechanism of miRNA non-seed sequence, many details in the mechanism remain far more elusive. For example, is the non-seed region-dependent transcriptional or post-transcriptional gene regulation a universal effect of miRNA? How many miRNAs have dual regulation on their targets like miR-1254? In terms of the components and functional mechanism of RISC, what’s the difference between non-seed sequence-modified and seed region modified-gene regulation? Addressing these questions will give us a further insight into miRNA-modified gene regulation and also a better understanding of miRNA functions in the oncogenic signaling network.

## Materials and methods

### Ethics statement

For studies using human data, the study was approved by the ethics committees of Shanghai Pulmonary Hospital (approval number: K17-136) and an informed consent was obtained from all participants.

For studies using animal data, all experiments were performed according to the National Institutes of Health Guide for the Care and Use of Laboratory Animals, the guidelines approved by the Institutional Animal Care and Use Committee of the Shanghai Institute of Materia Medica (approval number: 2017-01-RJ-136).

### Clinical cancer samples

All cancer samples were obtained from Shanghai pulmonary hospital (Shanghai, China) and were stored in liquid nitrogen until analysis. All experiments were conducted in accordance with the Declaration of Helsinki.

### Cell lines, culture conditions

Human lung adenocarcinoma cell lines (A549 and NCI-H1975) were obtained from the American Type Culture Collection (ATCC, USA). Cells were cultured in RPMI-1640 (Gibco, USA) medium supplemented with 10% fetal bovine serum (FBS). HEK293 cells were purchased from ATCC and cultured in DMEM medium supplemented with 10% FBS. Cells were maintained in a humidified incubator at 37°C with 5% CO_2_.

### Cell transfection

Transient transfection was performed using Lipofectamine 2000 (Invitrogen, Carlsbad, CA, USA) according to the manufacturer’s instructions. 50nM of small interfering RNA and 25nM miR-1254 mimic or antisense oligonucleotide was used. In the TFAP2A rescue experiment, 500 ng of plasmid DNA was used in a 6-well plate, and in the HO-1 rescue experiment, 100 ng of plasmid DNA was used in a 6-well plate.

### Plasmid constructs and RNA oligonucleotides

The plasmid pcDNA3.1-C5U (CCAR1 5`UTR) was a kind gift from Dr. Tao Zhu (School of Life Sciences, University of Science and Technology of China, Hefei, Anhui 230027, China). We cloned CCAR1 5`UTR into the lentiviral vector pCD513B-1 (pCD513B-1-1254) to over-express miR-1254. Human TFAP2A and HO-1 cDNA without 3′-UTR were cloned into pTT5 (obtained from Yves Durocher Lab) separately to construct the expression vectors. The 3`UTR of human HO-1 and TFAP2A was amplified via PCR using the genomic DNA of A549 and the PCR fragment was cloned into the psi-CHECK-2 vector separately (Promega, Madison, WI, USA). The promoter of human HO-1(~1.5kb) was also amplified via PCR using the genomic DNA of A549 and the PCR fragment was cloned into the PGL-4.10 vector. The primers used are listed in Supplementary S2 Table. All constructs were confirmed via DNA sequencing. miR-1254 mimic, anti-miR-1254 and small interfering RNAs targeting TFAP2A, or their respective negative control RNAs were purchased from GenePharma (Shanghai, China). The sequences of the RNA oligonucleotides are provided in Supplementary S1 Table.

### Site-directed mutant luciferase reporter plasmids

The mutated plasmid was cloned using the KODPlus-Mutagenesis Kit (Toyobo, Osaka, Japan) as previously reported[58]. All the primers were shown in Supplementary S2 Table. DNA sequencing confirmed the nucleotide sequence of these plasmids.

### RNA isolation and quantitative RT-PCR

Total RNA was extracted from cells using Trizol reagent (Invitrogen, USA) according to the manufacturer’s protocol. For HO-1 and TFAP2A expression, reverse transcription was performed with PrimeScript RT Master Mix (TaKaRa Biotechnology, China) following the manufacturer’s handbook. Quantitative real-time PCR (qPCR) was performed with QuantiNova SYBR Green PCR kit (Qiagen, USA) and analyzed on Rotor-Gene Q 2plex HRM System (Qiagen, USA).The relative HO-1 and TFAP2A mRNA levels were analyzed by normalizing the threshold cycle (Ct) value to that of internal loading control, β-actin. The primers are provided in the Supplementary S2 Table.

To quantify mature miR-1254, total RNA was reversely transcribed and amplified using TaqMan MicroRNA assay kit (Invitrogen, USA) according to the manufacturer's instructions. U6 snRNA were used as an internal loading control.

### Western blot

Total protein lysates were prepared from tumor cells and separated by 10% SDS-PAGE, transferred to PVDF membranes (Millipore, USA) and incubated with a primary antibody. HO-1 polyclonal antibody was purchased from Enzo Life Sciences, TFAP2A and PCNA antibodies were obtained from ABclonal Biotechnology, β-actin (Santa Cruze, USA) or α-tubulin (Cell Signaling Technology, Beverly, MA) was used as an internal control. The band densities were quantified by ImageQuant software (GE Healthcare, UK).

### Dual luciferase reporter assay

Cells seeded in 6-well plates were co-transfected with miR-1254 mimics (25 nM) or negative control and reporter constructs (200ng) using Lipofectamine 2000. Cell extracts were prepared 48h after transfection, and the luciferase activity was measured using the Dual-Luciferase Reporter Assay System (Promega).

### Cell counting and 3-(4, 5-dimethylthiazolyl-2-yl)-2-5 diphenyl tetrazolium bromide (MTT) assay

Cell number was measured using Vi-cell XR cell viability analyzer (Beckman coulter, USA). Cell viability was determined using MTT assay. Briefly, cells were harvested following 24 h of transfection and plated at 2 × 10^3^ cells per well in 96-well plates. After incubation, 20 μl MTT reagent (5.0 mg/mL) was added into each well and incubated in the dark at 37°C for 4 h. Then, 100 μl dissolution buffer was added into each well and incubated overnight. Absorbance was measured at 570 nm using a microtiter plate reader (Bio-Tek Instruments, USA).

### Colony formation assay

Twenty-four hours after transfection with miR-1254 mimics or negative control oligonucleotides, the NSCLC cells were seeded in 6-well plates and grew for two weeks for the colony formation assay. The cells were then washed with PBS, fixed with methyl alcohol, and stained by Gimsa and then photographed using Typhoon FLA 9500(GE Healthcare, UK). Colonies were counted by ImageQuant TL (GE Healthcare, UK).

### Annexin V-FITC apoptosis assay

Cells were transfected with 500 ng indicated plasmid DNA and 25nM miRNA oligonucleotides in a 6-well plate. Apoptotic cells were examined using an Annexin V-FITC Apoptosis Detection Kit (BD Biosciences, USA). The cells were harvested and then stained with 5 μl of annexin V-FITC and 5 μl of PI for 15 min at room temperature in the dark. The cells were measured by the BD FACS flow cytometer (BD Biosciences, USA).

### Chromatin immunoprecipitation

A549 cells (1 × 10^6^) were transfected with miR-1254 mimics or control oligonucleotides at a 25 nM final concentration. Forty-eight hours later, cells were cross-linked with 1% formaldehyde for 10 min at 37°C and chromatin immunoprecipitation (ChIP) assay was performed using the ChIP Assay Kit from Upstate (Millipore). Five micrograms of anti-RNA polymerase II antibody (Millipore) and anti-TFAP2A antibody (ABCam) were used for each assay. No antibody (input) and normal rabbit IgG (Santa Cruz) were used as controls. Quantitative real-time PCR data were normalized to chromatin input and expressed as fold changes relative to the values in the cells transfected with negative control RNA oligonucleotides (nc). Primers are listed in Supplementary S2 Table.

### CRISPR/Cas9-modified nucleotides knockdown

The px330-mCherry and px330-GFP vectors were a kind gift from Dr. Hui Yang (Institute of neuroscience, Chinese academy of sciences, Shanghai, China) CRISPR/Cas9-modified nucleotide deletion was performed as previously described [28, 59]. Two sgRNAs were cloned into px330-mCherry and px330-GFP vectors, respectively. The sgRNA sequences are as follow: CRISPR-1254-left, 5`-caccgCCCAGCTACTTGGGAAGCTG-3`; CRISPR-1254-right, 5`-caccGTGTGTGTAAGGTTGCAGCT-3`; CRISPR-sites-left, 5`-caccgCACACCCCTGTGCCCTCATG-3`; CRISPR-sites-right, 5`-caccgACGGCCTGTTCTGTTCTCTT-3`. The plasmids were co-transfected into A549 cells (1 μg each) in a 6-well plate, and positively transfected cells were isolated using flow cytometry. The genome modification of each single cell used in the following studies was confirmed via DNA sequencing. Primers are listed in Supplementary S2 Table

### mRNA decay measurements

To estimate the mRNA decay rates, transcription was inhibited by adding 5 μg/ml actinomycin D in medium [41, 42]. RNA was extracted at the indicated times and analyzed by qRT-PCR. The ratio of HO-1 mRNA to β-actin in each sample was calculated and used to determine the relative amount of specific mRNA remaining in each sample.

### Animal study

Animal studies were performed according to the National Institutes of Health Guide for the Care and Use of Laboratory Animals. Stable miR-1254–overexpressing A549 cells (A549/miR-1254) were harvested by trypsin, washed with PBS, and resuspended in Matrigel:RPMI medium (1:1); 1 million A549/miR-1254 cells and corresponding control cells were subcutaneously injected into the nude mice. Tumor volumes were calculated from the length (a) and the width (b) by using the following formula: volume (millimeters^3^) = ab^2^/2.

### Statistical analyses

All statistical analyses were performed using GraphPad Prism software (version 5.01; GraphPad Software, Inc, CA, USA). The data are shown as the mean values with standard error of mean (SEM), and P<0.05 was considered significant. All experiments were performed independently at least three times. The significance of differences between two groups was measured by Student’s t test. One-way analysis of variance (ANOVA) was used to measure the significance of comparisons between more than two groups.

## Supporting information

S1 Fig The effect of miR-1254 mutants on HO-1 promoter activity. Luciferase activity in HEK293 cells transfected with the indicated nucleotides (MiR-1254 and its 5`mt) for 48 h.
Data are presented as the mean ± SEM of three independent experiments. *P<0.05, **P and ***P <0.01 vs. nc; #*P*<0.05, ##*P* and ###*P* <0.01 vs miR-1254.
(TIF)

S2 Fig The exploration of miR-1254 potential binding site on HO-1 promoter. (A) Upper: Schematic representation of miR-1254 potential binding sites analyzed by miRBase database and RNA hybrid; Bottom: Non-denaturing PAGE experiment is performed to test the binding ability of miR-1254 with these sites.
(B) Schematic representation of the CRISPR strategy for site2 deletion.
(C) qRT-PCR measurement of the effect of miR-1254 on HO-1 mRNA expression in the wild type (WT) and site 2 deleted (MT) cell lines.
(TIF)

S3 Fig miR-1254 inhibits HO-1 expression not by directly targets HO-1 promoter or induces DNA methylation. (A) Construction of six luciferase reporters containing varying length fragments of HO-1 promoter.
(B) Luciferase activity of miR-1254 on the six HO-1 promoter plasmids in HEK293 cells.
(C) Luciferase activity of miR-1254 on the wild-type and the mutated HO-1 promoter PGL-HO1.
(D) qRT-PCR analysis of the mRNA level of HO-1 in A549 cells after transfection of miR-1254 with 1μM Decitabine.
Data are presented as the mean ± SEM of three independent experiments. *P<0.05, **P and ***P <0.01 vs. nc; ###*P* <0.01 vs miR-1254.
(TIF)

S4 Fig miR-1254 down-regulates HO-1 transcription via targeting TFAP2A in NCI-H1975 cells. (A) HO-1 and TFAP2A mRNA levels in NCI-H1975 cells transfected with the indicated nucleotides (MiR-1254 and its mutants) for 48 h assayed by qRT-PCR.
(B) Western blot analysis of the effect of TFAP2A knockdown on HO-1 protein expression compared with miR-1254 in NCI-H1975 cells.
(C) TFAP2A and HO-1 protein levels in NCI-H1975 cells transfected with the indicated nucleotides (miR-1254 and its mutants) for 48 h assayed by immunoblotting.
(D) Ectopic expression of TFAP2A overrode the inhibition of HO-1 expression by miR-1254 in NCI-H1975 cells.
(E) Statistical results of HO-1 protein when TFAP2A cDNA co-transfected with miR-1254.
Data are presented as the mean ± SEM of three independent experiments. *P<0.05, **P<0.01 vs. nc; ##*P* and ###*P* <0.01 vs miR-1254.
(TIF)

S5 Fig MiR-1254 inhibited the cell growth of NCI-H1975 cells. (A-C) MiR-1254 inhibited the cell growth of NCI-H1975 cells. Cells were transfected with miR-1254 mimics or negative control oligonuleotides (nc), 20μM hemin was used to rescue the expression of HO-1 as a inducer.
(A) Trypan blue staining assays. Cells were counted 72h after transfection.
(B) MTT analysis of NCI-H1975 cells transfected with miR-1254 mimics or nc.
(C) Colony formation in NCI-H1975 cells transfected with miR-1254 mimics compared with nc. Upper: Representative image of the colony formation. Bottom: Statistical results.
(D and E) Flow cytometry analysis of cell cycle (D) and apoptosis (E) in NCI-H1975 cells.
(F) Upper: MiR-1254 expression in A549/miR-1254 and A549/miR-Control cells. Bottom: Western blot analysis of the TFAP2A and HO-1 protein levels in the A549/miR-1254 cells and A549/miR-Control cells.
Data are presented as the mean ± SEM of three independent experiments. *P<0.05, **P and ***P <0.01 vs. nc; #*P*<0.05 and ###*P* <0.01 vs miR-1254.
(TIF)

S1 Table Sequence of siRNA and miRNA mimics used in this study. (DOCX)

S2 Table Sequence of qRT-PCR and PCR primers used in this study. (DOCX)

## References

[1] Liu K, Li X, Cao Y, Ge Y, Wang J, Shi B. miR-132 inhibits cell proliferation, invasion and migration of hepatocellular carcinoma by targeting PIK3R3. Int J Oncol. 2015 doi: 10.3892/ijo.2015.3112 . 26252738 10.3892/ijo.2015.3112

[2] Liu N, Olson EN. MicroRNA regulatory networks in cardiovascular development. Dev Cell. 2010;18(4):510–25. doi: 10.1016/j.devcel.2010.03.010 ; PubMed Central PMCID: PMCPMC2922691. 20412767 10.1016/j.devcel.2010.03.010 PMC2922691

[3] Sun M, Huang F, Yu D, Zhang Y, Xu H, Zhang L, et al Autoregulatory loop between TGF-beta1/miR-411-5p/SPRY4 and MAPK pathway in rhabdomyosarcoma modulates proliferation and differentiation. Cell Death Dis. 2015;6:e1859 doi: 10.1038/cddis.2015.225 . 26291313 10.1038/cddis.2015.225 PMC4558514

[4] He XH, Zhu W, Yuan P, Jiang S, Li D, Zhang HW, et al miR-155 downregulates ErbB2 and suppresses ErbB2-induced malignant transformation of breast epithelial cells. Oncogene. 2016;35(46):6015–25. doi: 10.1038/onc.2016.132 . 27065318 10.1038/onc.2016.132

[5] Shenoy A, Danial M, Blelloch RH. Let-7 and miR-125 cooperate to prime progenitors for astrogliogenesis. EMBO J. 2015;34(9):1180–94. doi: 10.15252/embj.201489504 ; PubMed Central PMCID: PMCPMC4426479. 25715649 10.15252/embj.201489504 PMC4426479

[6] Cao Y, Guo WT, Tian S, He X, Wang XW, Liu X, et al miR-290/371-Mbd2-Myc circuit regulates glycolytic metabolism to promote pluripotency. EMBO J. 2015;34(5):609–23. doi: 10.15252/embj.201490441 ; PubMed Central PMCID: PMCPMC4365031. 25603933 10.15252/embj.201490441 PMC4365031

[7] Sun HL, Cui R, Zhou J, Teng KY, Hsiao YH, Nakanishi K, et al ERK Activation Globally Downregulates miRNAs through Phosphorylating Exportin-5. Cancer Cell. 2016;30(5):723–36. doi: 10.1016/j.ccell.2016.10.001 ; PubMed Central PMCID: PMCPMC5127275. 27846390 10.1016/j.ccell.2016.10.001 PMC5127275

[8] Tie J, Pan Y, Zhao L, Wu K, Liu J, Sun S, et al MiR-218 inhibits invasion and metastasis of gastric cancer by targeting the Robo1 receptor. PLoS Genet. 2010;6(3):e1000879 doi: 10.1371/journal.pgen.1000879 ; PubMed Central PMCID: PMCPMC2837402. 20300657 10.1371/journal.pgen.1000879 PMC2837402

[9] Ge Y, Yan X, Jin Y, Yang X, Yu X, Zhou L, et al fMiRNA-192 and miRNA-204 Directly Suppress lncRNA HOTTIP and Interrupt GLS1-Modified Glutaminolysis in Hepatocellular Carcinoma. PLoS Genet. 2015;11(12):e1005726 doi: 10.1371/journal.pgen.1005726 ; PubMed Central PMCID: PMCPMC4692503. 26710269 10.1371/journal.pgen.1005726 PMC4692503

[10] Bartel DP. MicroRNAs: target recognition and regulatory functions. Cell. 2009;136(2):215–33. doi: 10.1016/j.cell.2009.01.002 ; PubMed Central PMCID: PMCPMC3794896. 19167326 10.1016/j.cell.2009.01.002 PMC3794896

[11] Seok H, Ham J, Jang ES, Chi SW. MicroRNA Target Recognition: Insights from Transcriptome-Wide Non-Canonical Interactions. Mol Cells. 2016;39(5):375–81. doi: 10.14348/molcells.2016.0013 ; PubMed Central PMCID: PMCPMC4870184. 27117456 10.14348/molcells.2016.0013 PMC4870184

[12] Friedman RC, Farh KKH, Burge CB, Bartel DP. Most mammalian mRNAs are conserved targets of microRNAs. Genome Research. 2009;19(1):92–105. doi: 10.1101/gr.082701.108 18955434 10.1101/gr.082701.108 PMC2612969

[13] Grimson A, Farh KK, Johnston WK, Garrett-Engele P, Lim LP, Bartel DP. MicroRNA targeting specificity in mammals: determinants beyond seed pairing. Mol Cell. 2007;27(1):91–105. doi: 10.1016/j.molcel.2007.06.017 ; PubMed Central PMCID: PMCPMC3800283. 17612493 10.1016/j.molcel.2007.06.017 PMC3800283

[14] Yekta S, Shih IH, Bartel DP. MicroRNA-directed cleavage of HOXB8 mRNA. Science. 2004;304(5670):594–6. doi: 10.1126/science.1097434 15105502 10.1126/science.1097434

[15] Vella MC, Choi EY, Lin SY, Reinert K, Slack FJ. The C. elegans microRNA let-7 binds to imperfect let-7 complementary sites from the lin-41 3 ' UTR. Gene Dev. 2004;18(2):132–7. doi: 10.1101/gad.1165404 14729570 10.1101/gad.1165404 PMC324419

[16] Brennecke J, Stark A, Russell RB, Cohen SM. Principles of microRNA-target recognition. PLoS Biol. 2005;3(3):e85 doi: 10.1371/journal.pbio.0030085 ; PubMed Central PMCID: PMCPMC1043860. 15723116 10.1371/journal.pbio.0030085 PMC1043860

[17] Chi SW, Zang JB, Mele A, Darnell RB. Argonaute HITS-CLIP decodes microRNA-mRNA interaction maps. Nature. 2009;460(7254):479–86. doi: 10.1038/nature08170 19536157 10.1038/nature08170 PMC2733940

[18] Chi SW, Hannon GJ, Darnell RB. An alternative mode of microRNA target recognition. Nature Structural & Molecular Biology. 2012;19(3):321–U80. 10.1038/nsmb.2230 PMC3541676 22343717

[19] Shin C, Nam JW, Farh KK, Chiang HR, Shkumatava A, Bartel DP. Expanding the microRNA targeting code: functional sites with centered pairing. Mol Cell. 2010;38(6):789–802. doi: 10.1016/j.molcel.2010.06.005 ; PubMed Central PMCID: PMCPMC2942757. 20620952 10.1016/j.molcel.2010.06.005 PMC2942757

[20] Martin HC, Wani S, Steptoe AL, Krishnan K, Nones K, Nourbakhsh E, et al Imperfect centered miRNA binding sites are common and can mediate repression of target mRNAs. Genome Biol. 2014;15(3). 10.1186/gb-2014-15-3-r51 PMC4053950 24629056

[21] Kim DH, Saetrom P, Snove O, Rossi JJ. MicroRNA-directed transcriptional gene silencing in mammalian cells. P Natl Acad Sci USA. 2008;105(42):16230–5. 10.1073/pnas.0808830105 PMC2571020 18852463

[22] Younger ST, Corey DR. Transcriptional gene silencing in mammalian cells by miRNA mimics that target gene promoters. Nucleic Acids Res. 2011;39(13):5682–91. doi: 10.1093/nar/gkr155 ; PubMed Central PMCID: PMCPMC3141263. 21427083 10.1093/nar/gkr155 PMC3141263

[23] Benhamed M, Herbig U, Ye T, Dejean A, Bischof O. Senescence is an endogenous trigger for microRNA-directed transcriptional gene silencing in human cells. Nat Cell Biol. 2012;14(3):266–75. doi: 10.1038/ncb2443 . 22366686 10.1038/ncb2443 PMC5423543

[24] Zardo G, Ciolfi A, Vian L, Starnes LM, Billi M, Racanicchi S, et al Polycombs and microRNA-223 regulate human granulopoiesis by transcriptional control of target gene expression. Blood. 2012;119(17):4034–46. doi: 10.1182/blood-2011-08-371344 . 22327224 10.1182/blood-2011-08-371344

[25] Tan Y, Zhang B, Wu T, Skogerbo G, Zhu X, Guo X, et al Transcriptional inhibiton of Hoxd4 expression by miRNA-10a in human breast cancer cells. BMC Mol Biol. 2009;10:12 doi: 10.1186/1471-2199-10-12 ; PubMed Central PMCID: PMCPMC2680403. 19232136 10.1186/1471-2199-10-12 PMC2680403

[26] Adilakshmi T, Sudol I, Tapinos N. Combinatorial Action of miRNAs Regulates Transcriptional and Post-Transcriptional Gene Silencing following in vivo PNS Injury. Plos One. 2012;7(7). 10.1371/journal.pone.0039674 PMC3391190 22792185

[27] Roberts TC. The MicroRNA Biology of the Mammalian Nucleus. Mol Ther Nucleic Acids. 2014;3:e188 doi: 10.1038/mtna.2014.40 ; PubMed Central PMCID: PMCPMC4221600. 25137140 10.1038/mtna.2014.40 PMC4221600

[28] Miao L, Yao H, Li C, Pu M, Yao X, Yang H, et al A dual inhibition: microRNA-552 suppresses both transcription and translation of cytochrome P450 2E1. Biochim Biophys Acta. 2016;1859(4):650–62. doi: 10.1016/j.bbagrm.2016.02.016 . 26926595 10.1016/j.bbagrm.2016.02.016

[29] Tenhunen R, Marver HS, Schmid R. The enzymatic conversion of heme to bilirubin by microsomal heme oxygenase. Proc Natl Acad Sci U S A. 1968;61(2):748–55. ; PubMed Central PMCID: PMCPMC225223. 4386763 10.1073/pnas.61.2.748 PMC225223

[30] Tenhunen R MH, Schmid R. Microsomal heme oxygenase. Characterization of the enzyme.pdf. 1969 12 10;244(23):6388–94. 4390967

[31] Torisu-Itakura H, Furue M, Kuwano M, Ono M. Co-expression of thymidine phosphorylase and heme oxygenase-1 in macrophages in human malignant vertical growth melanomas. Jpn J Cancer Res. 2000;91(9):906–10. Epub 2000/09/30. . 11011118 10.1111/j.1349-7006.2000.tb01033.x PMC5926440

[32] Deininger MH, Meyermann R, Trautmann K, Duffner F, Grote EH, Wickboldt J, et al Heme oxygenase (HO)-1 expressing macrophages/microglial cells accumulate during oligodendroglioma progression. Brain Res. 2000;882(1–2):1–8. . 11056178 10.1016/s0006-8993(00)02594-4

[33] Berberat PO, Dambrauskas Z, Gulbinas A, Giese T, Giese N, Kunzli B, et al Inhibition of heme oxygenase-1 increases responsiveness of pancreatic cancer cells to anticancer treatment. Clin Cancer Res. 2005;11(10):3790–8. doi: 10.1158/1078-0432.CCR-04-2159 . 15897578 10.1158/1078-0432.CCR-04-2159

[34] Maines MD, Abrahamsson PA. Expression of heme oxygenase-1 (HSP32) in human prostate: normal, hyperplastic, and tumor tissue distribution. Urology. 1996;47(5):727–33. . 8650873 10.1016/s0090-4295(96)00010-6

[35] Degese MS, Mendizabal JE, Gandini NA, Gutkind JS, Molinolo A, Hewitt SM, et al Expression of heme oxygenase-1 in non-small cell lung cancer (NSCLC) and its correlation with clinical data. Lung Cancer. 2012;77(1):168–75. Epub 2012/03/16. doi: 10.1016/j.lungcan.2012.02.016 . 22418244 10.1016/j.lungcan.2012.02.016 PMC8381257

[36] Jozkowicz A, Was H, Dulak J. Heme oxygenase-1 in tumors: is it a false friend? Antioxid Redox Signal. 2007;9(12):2099–117. doi: 10.1089/ars.2007.1659 ; PubMed Central PMCID: PMCPMC2096718. 17822372 10.1089/ars.2007.1659 PMC2096718

[37] Li CG, Pu MF, Li CZ, Gao M, Liu MX, Yu CZ, et al MicroRNA-1304 suppresses human non-small cell lung cancer cell growth in vitro by targeting heme oxygenase-1. Acta Pharmacol Sin. 2017;38(1):110–9. doi: 10.1038/aps.2016.92 ; PubMed Central PMCID: PMCPMC5220548. 27641735 10.1038/aps.2016.92 PMC5220548

[38] Lewis BP, Burge CB, Bartel DP. Conserved seed pairing, often flanked by adenosines, indicates that thousands of human genes are microRNA targets. Cell. 2005;120(1):15–20. doi: 10.1016/j.cell.2004.12.035 . 15652477 10.1016/j.cell.2004.12.035

[39] Griffiths-Jones S, Grocock RJ, van Dongen S, Bateman A, Enright AJ. miRBase: microRNA sequences, targets and gene nomenclature. Nucleic Acids Res. 2006;34(Database issue):D140–4. doi: 10.1093/nar/gkj112 ; PubMed Central PMCID: PMCPMC1347474. 16381832 10.1093/nar/gkj112 PMC1347474

[40] Beckman JD, Chen CS, Nguyen J, Thayanithy V, Subramanian S, Steer CJ, et al Regulation of Heme Oxygenase-1 Protein Expression by miR-377 in Combination with miR-217. Journal of Biological Chemistry. 2011;286(5):3194–202. doi: 10.1074/jbc.M110.148726 21106538 10.1074/jbc.M110.148726 PMC3030323

[41] von Knethen A, Neb H, Morbitzer V, Schmidt MV, Kuhn AM, Kuchler L, et al PPARgamma stabilizes HO-1 mRNA in monocytes/macrophages which affects IFN-beta expression. Free Radic Biol Med. 2011;51(2):396–405. doi: 10.1016/j.freeradbiomed.2011.04.033 . 21571064 10.1016/j.freeradbiomed.2011.04.033

[42] Emerald BS, Chen Y, Zhu T, Zhu Z, Lee KO, Gluckman PD, et al AlphaCP1 mediates stabilization of hTERT mRNA by autocrine human growth hormone. J Biol Chem. 2007;282(1):680–90. doi: 10.1074/jbc.M600224200 . 17085453 10.1074/jbc.M600224200

[43] Kruger J, Rehmsmeier M. RNAhybrid: microRNA target prediction easy, fast and flexible. Nucleic Acids Research. 2006;34:W451–W4. doi: 10.1093/nar/gkl243 16845047 10.1093/nar/gkl243 PMC1538877

[44] Kawasaki H TK. Induction of DNA methylation and gene silencing by short interfering RNAs in human cells. Nature. 2004;431(7005):205–11. doi: 10.1038/nature02889 . 15311210 10.1038/nature02889

[45] Zhang Y, Cheng C, He D, Shi W, Fu C, Wang X, et al Transcriptional gene silencing of dopamine D3 receptor caused by let-7d mimics in immortalized renal proximal tubule cells of rats. Gene. 2016;580(2):89–95. doi: 10.1016/j.gene.2015.12.071 . 26802971 10.1016/j.gene.2015.12.071

[46] Loboda A, Jazwa A, Grochot-Przeczek A, Rutkowski AJ, Cisowski J, Agarwal A, et al Heme oxygenase-1 and the vascular bed: from molecular mechanisms to therapeutic opportunities. Antioxid Redox Signal. 2008;10(10):1767–812. doi: 10.1089/ars.2008.2043 . 18576916 10.1089/ars.2008.2043

[47] Alam J, Cook JL. How many transcription factors does it take to turn on the heme oxygenase-1 gene? Am J Respir Cell Mol Biol. 2007;36(2):166–74. doi: 10.1165/rcmb.2006-0340TR . 16990612 10.1165/rcmb.2006-0340TR

[48] Alam J, Den Z. Distal AP-1 binding sites mediate basal level enhancement and TPA induction of the mouse heme oxygenase-1 gene. J Biol Chem. 1992;267(30):21894–900. . 1400499

[49] Nascimento AL, Luscher P, Tyrrell RM. Ultraviolet A (320–380 nm) radiation causes an alteration in the binding of a specific protein/protein complex to a short region of the promoter of the human heme oxygenase 1 gene. Nucleic Acids Res. 1993;21(5):1103–9. ; PubMed Central PMCID: PMCPMC309269. 7840819 10.1093/nar/21.5.1103 PMC309269

[50] Takahashi S, Takahashi Y, Ito K, Nagano T, Shibahara S, Miura T. Positive and negative regulation of the human heme oxygenase-1 gene expression in cultured cells. Biochim Biophys Acta. 1999;1447(2–3):231–5. . 10542320 10.1016/s0167-4781(99)00156-6

[51] Li G, Wu X, Qian W, Cai H, Sun X, Zhang W, et al CCAR1 5' UTR as a natural miRancer of miR-1254 overrides tamoxifen resistance. Cell Res. 2016;26(6):655–73. doi: 10.1038/cr.2016.32 ; PubMed Central PMCID: PMCPMC4897177. 27002217 10.1038/cr.2016.32 PMC4897177

[52] Venkatesan N, Deepa PR, Khetan V, Krishnakumar S. Computational and in vitro Investigation of miRNA-Gene Regulations in Retinoblastoma Pathogenesis: miRNA Mimics Strategy. Bioinform Biol Insights. 2015;9:89–101. doi: 10.4137/BBI.S21742 ; PubMed Central PMCID: PMCPMC4429751. 25983556 10.4137/BBI.S21742 PMC4429751

[53] Foss KM, Sima C, Ugolini D, Neri M, Allen KE, Weiss GJ. miR-1254 and miR-574-5p Serum-Based microRNA Biomarkers for Early-Stage Non-small Cell Lung Cancer. J Thorac Oncol. 2011;6(3):482–8. doi: 10.1097/JTO.0b013e318208c785 21258252 10.1097/JTO.0b013e318208c785

[54] Peng H, Wang J, Li J, Zhao M, Huang SK, Gu YY, et al A circulating non-coding RNA panel as an early detection predictor of non-small cell lung cancer. Life Sci. 2016;151:235–42. doi: 10.1016/j.lfs.2016.03.002 . 26946307 10.1016/j.lfs.2016.03.002

[55] Chau LY. Heme oxygenase-1: emerging target of cancer therapy. Journal of biomedical science. 2015;22:22 doi: 10.1186/s12929-015-0128-0 ; PubMed Central PMCID: PMC4380252. 25885228 10.1186/s12929-015-0128-0 PMC4380252

[56] Surh YJ. Cancer chemoprevention with dietary phytochemicals. Nature reviews Cancer. 2003;3(10):768–80. doi: 10.1038/nrc1189 . 14570043 10.1038/nrc1189

[57] Gao C, Peng FH, Peng LK. MiR-200c sensitizes clear-cell renal cell carcinoma cells to sorafenib and imatinib by targeting heme oxygenase-1. Neoplasma. 2014;61(6):680–9. doi: 10.4149/neo_2014_083 25150313 10.4149/neo_2014_083

[58] Gao M. miR-145 sensitizes breast cancer to doxorubicin by targeting multidrug resistance-associated protein-1.pdf. Oncotarget. 2016. 10.18632/oncotarget.10845 PMC5312343 27487127

[59] Yang H, Wang H, Shivalila CS, Cheng AW, Shi L, Jaenisch R. One-step generation of mice carrying reporter and conditional alleles by CRISPR/Cas-modified genome engineering. Cell. 2013;154(6):1370–9. doi: 10.1016/j.cell.2013.08.022 ; PubMed Central PMCID: PMCPMC3961003. 23992847 10.1016/j.cell.2013.08.022 PMC3961003

