# Supplementary figures and images for: MiR-1254 suppresses HO-1 expression through seed region-dependent silencing and non-seed interaction with TFAP2A transcript to attenuate NSCLC growth
Source: PLoS Genet. 2017 Jul 27;13(7):e1006896. doi: 10.1371/journal.pgen.1006896 (PMC5549757; doi:10.1371/journal.pgen.1006896)

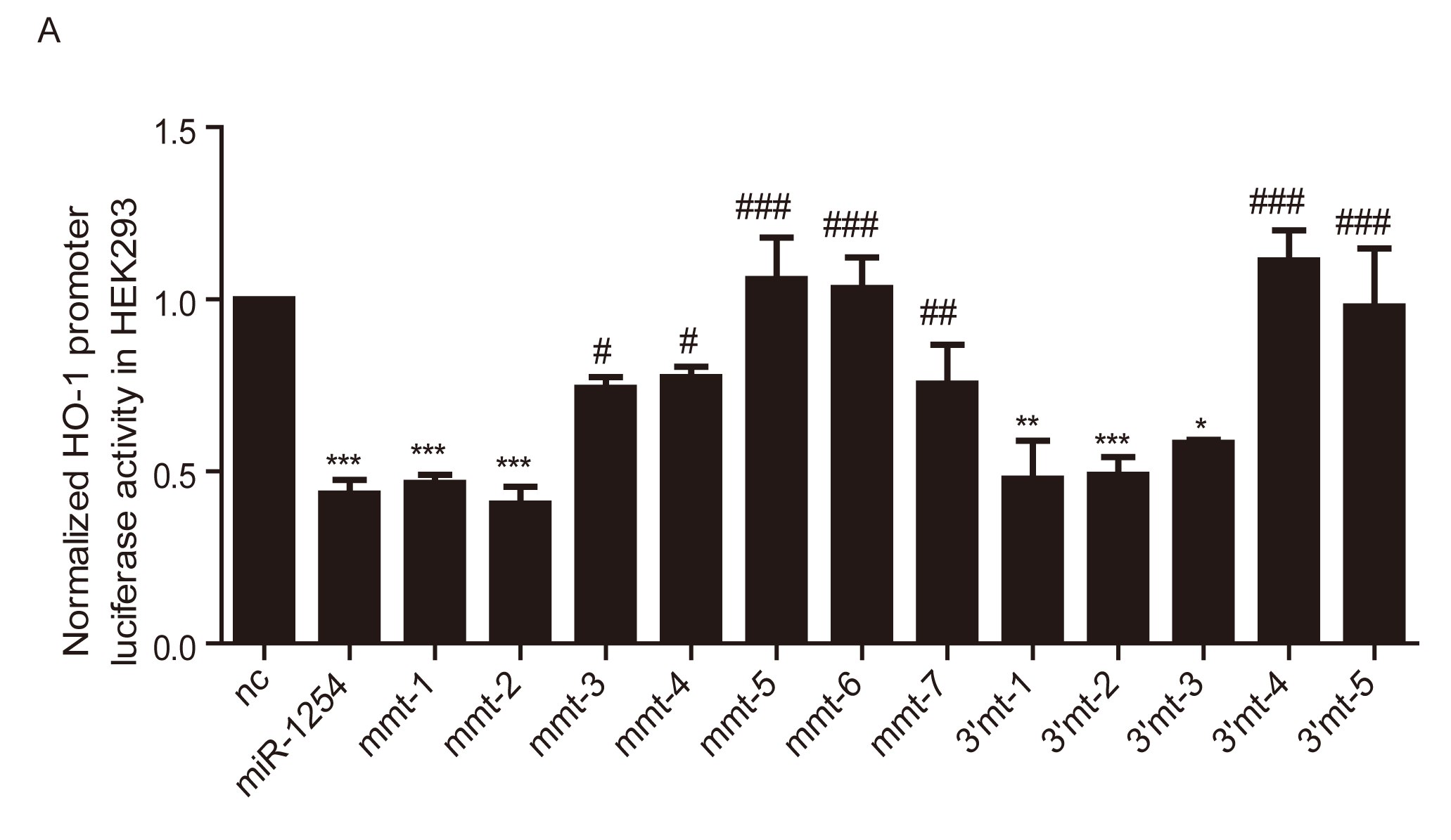

Supplement: S1 Fig — Luciferase activity in HEK293 cells transfected with the indicated nucleotides (MiR-1254 and its 5`mt) for 48 h. Data are presented as the mean ± SEM of three independent experiments. *P<0.05, **P and ***P <0.01 vs. nc; #P<0.05, ##P and ###P <0.01 vs miR-1254. (TIF) [file pgen.1006896.s001.tif]

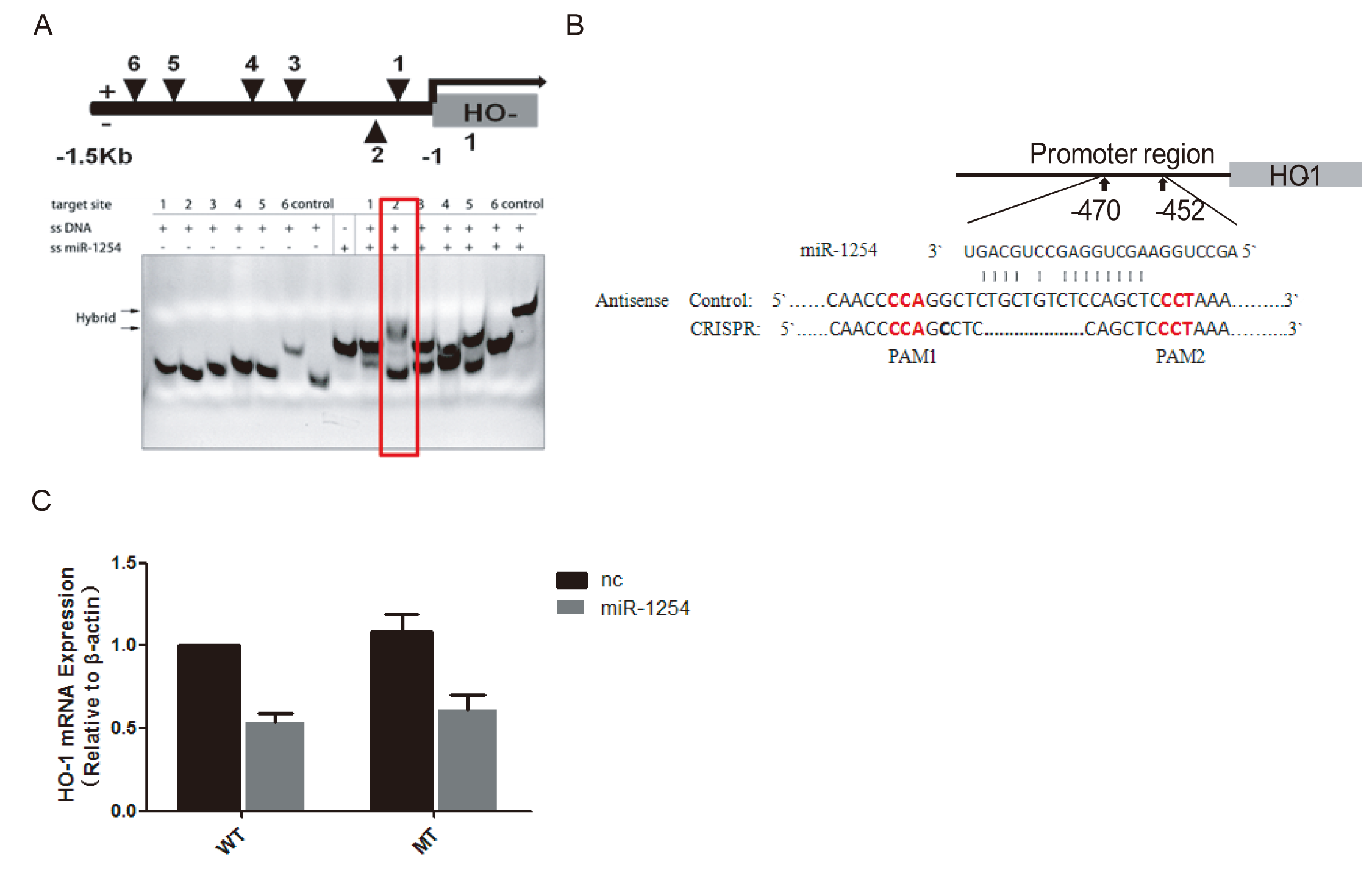

Supplement: S2 Fig — (A) Upper: Schematic representation of miR-1254 potential binding sites analyzed by miRBase database and RNA hybrid; Bottom: Non-denaturing PAGE experiment is performed to test the binding ability of miR-1254 with these sites. (B) Schematic representation of the CRISPR strategy for site2 deletion. (C) qRT-PCR measurement of the effect of miR-1254 on HO-1 mRNA expression in the wild type (WT) and site 2 deleted (MT) cell lines. (TIF) [file pgen.1006896.s002.tif]

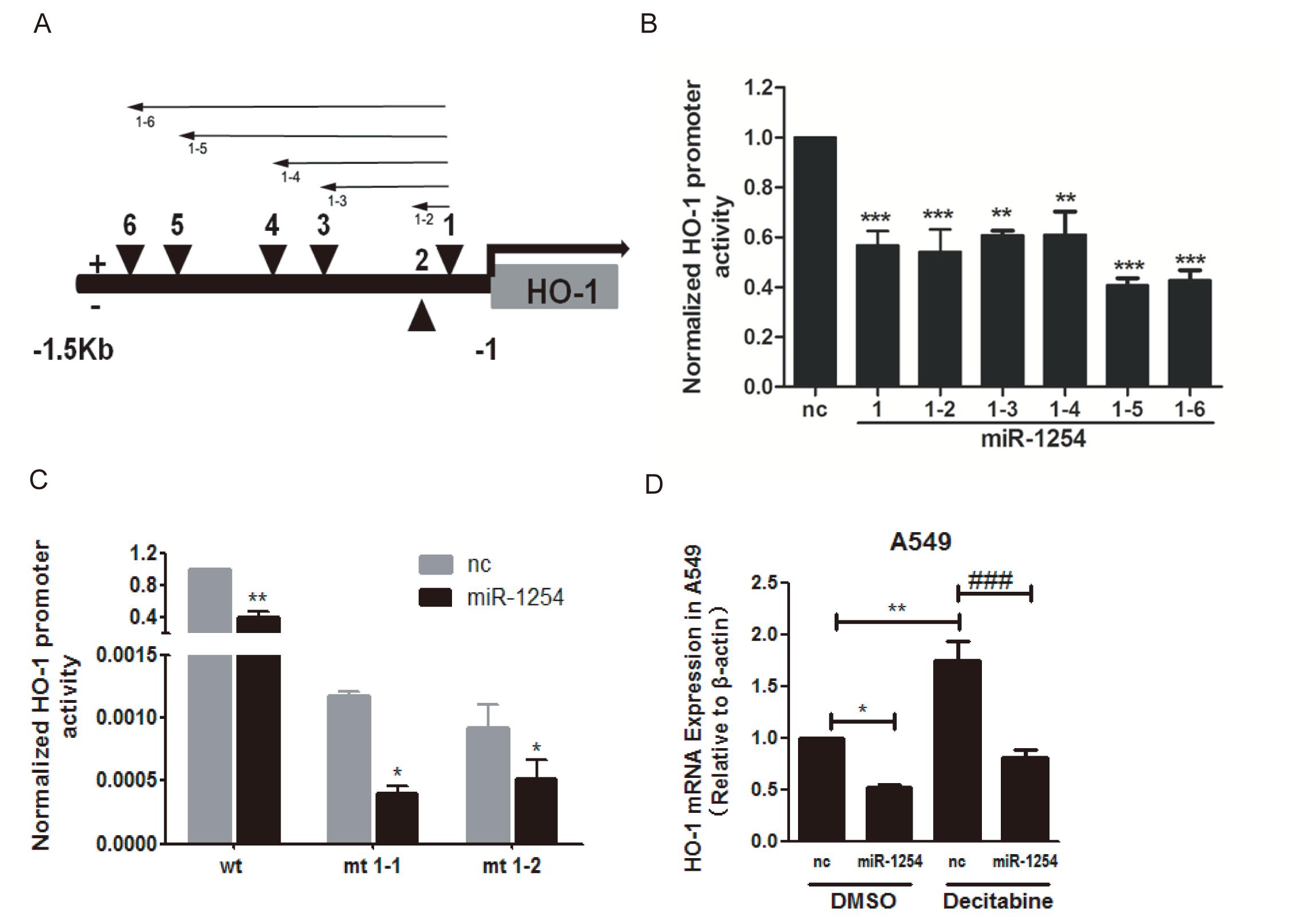

Supplement: S3 Fig — (A) Construction of six luciferase reporters containing varying length fragments of HO-1 promoter. (B) Luciferase activity of miR-1254 on the six HO-1 promoter plasmids in HEK293 cells. (C) Luciferase activity of miR-1254 on the wild-type and the mutated HO-1 promoter PGL-HO1. (D) qRT-PCR analysis of the mRNA level of HO-1 in A549 cells after transfection of miR-1254 with 1μM Decitabine. Data are presented as the mean ± SEM of three independent experiments. *P<0.05, **P and ***P <0.01 vs. nc; ###P <0.01 vs miR-1254. (TIF) [file pgen.1006896.s003.tif]

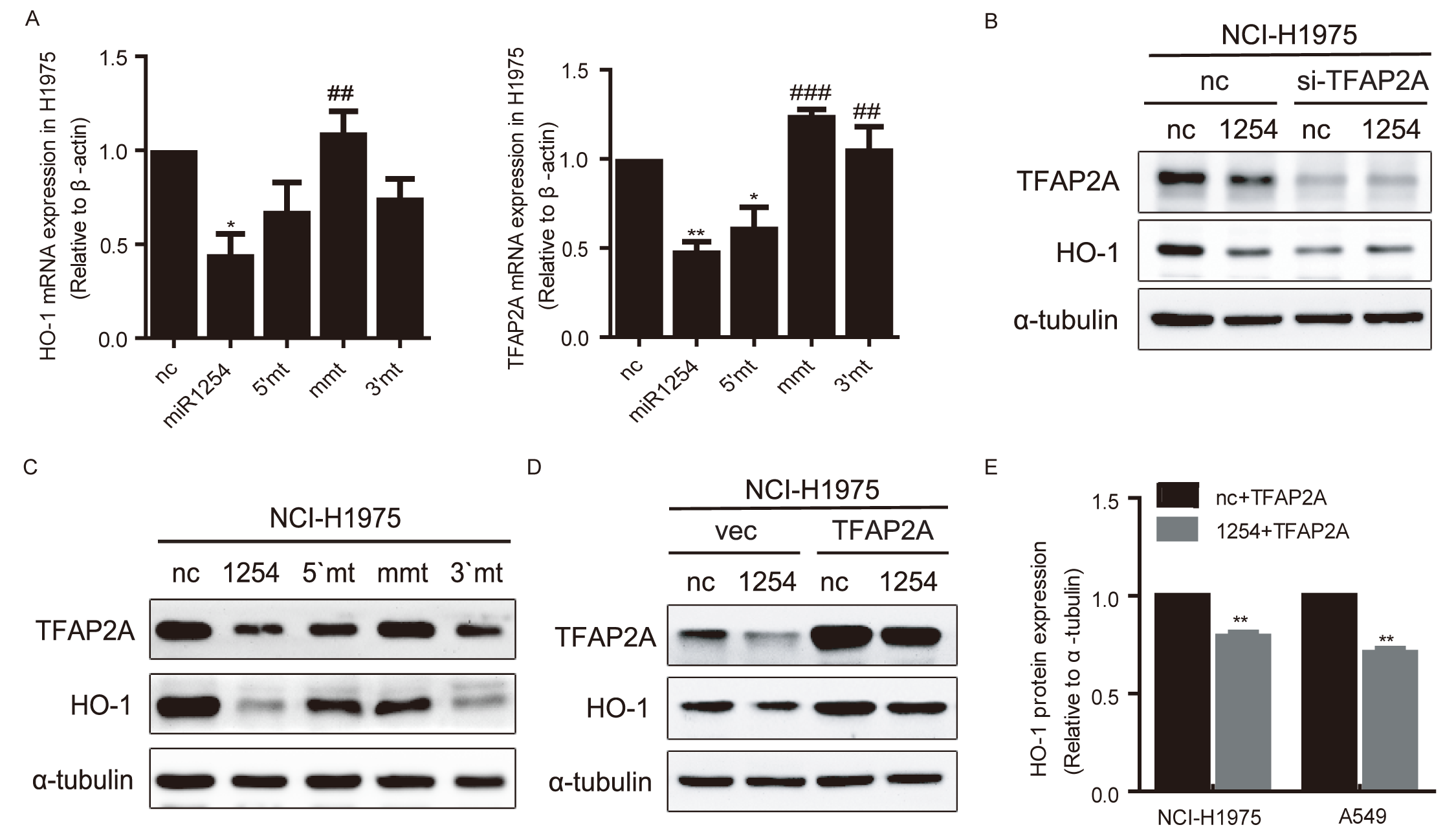

Supplement: S4 Fig — (A) HO-1 and TFAP2A mRNA levels in NCI-H1975 cells transfected with the indicated nucleotides (MiR-1254 and its mutants) for 48 h assayed by qRT-PCR. (B) Western blot analysis of the effect of TFAP2A knockdown on HO-1 protein expression compared with miR-1254 in NCI-H1975 cells. (C) TFAP2A and HO-1 protein levels in NCI-H1975 cells transfected with the indicated nucleotides (miR-1254 and its mutants) for 48 h assayed by immunoblotting. (D) Ectopic expression of TFAP2A overrode the inhibition of HO-1 expression by miR-1254 in NCI-H1975 cells. (E) Statistical results of HO-1 protein when TFAP2A cDNA co-transfected with miR-1254. Data are presented as the mean ± SEM of three independent experiments. *P<0.05, **P<0.01 vs. nc; ##P and ###P <0.01 vs miR-1254. (TIF) [file pgen.1006896.s004.tif]

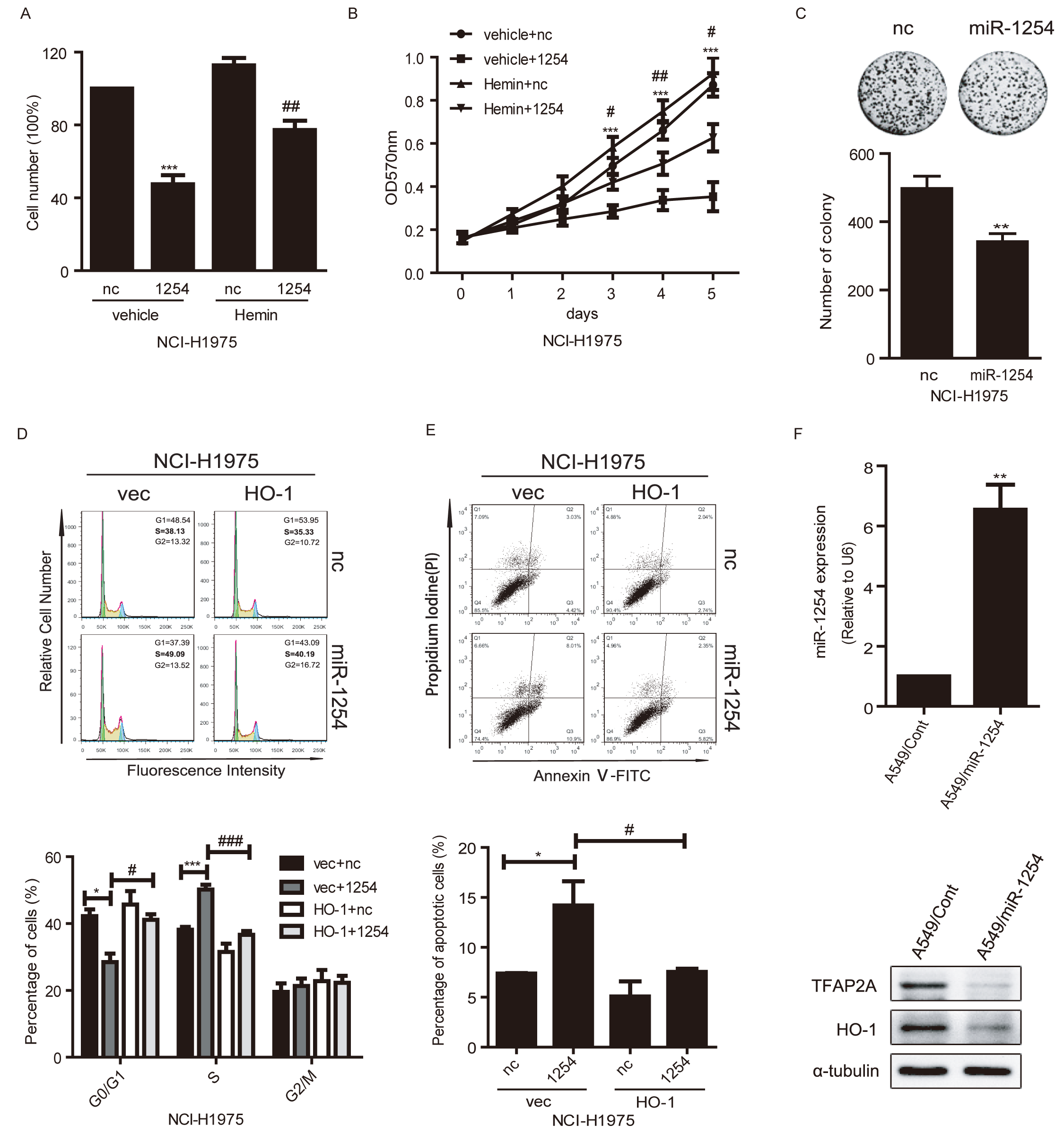

Supplement: S5 Fig — (A-C) MiR-1254 inhibited the cell growth of NCI-H1975 cells. Cells were transfected with miR-1254 mimics or negative control oligonuleotides (nc), 20μM hemin was used to rescue the expression of HO-1 as a inducer. (A) Trypan blue staining assays. Cells were counted 72h after transfection. (B) MTT analysis of NCI-H1975 cells transfected with miR-1254 mimics or nc. (C) Colony formation in NCI-H1975 cells transfected with miR-1254 mimics compared with nc. Upper: Representative image of the colony formation. Bottom: Statistical results. (D and E) Flow cytometry analysis of cell cycle (D) and apoptosis (E) in NCI-H1975 cells. (F) Upper: MiR-1254 expression in A549/miR-1254 and A549/miR-Control cells. Bottom: Western blot analysis of the TFAP2A and HO-1 protein levels in the A549/miR-1254 cells and A549/miR-Control cells. Data are presented as the mean ± SEM of three independent experiments. *P<0.05, **P and ***P <0.01 vs. nc; #P<0.05 and ###P <0.01 vs miR-1254. (TIF) [file pgen.1006896.s005.tif]
