# Supplementary material for: MiR-1254 suppresses HO-1 expression through seed region-dependent silencing and non-seed interaction with TFAP2A transcript to attenuate NSCLC growth
Source: PLoS Genet. 2017 Jul 27;13(7):e1006896. doi: 10.1371/journal.pgen.1006896 (PMC5549757; doi:10.1371/journal.pgen.1006896)
Supplement: S1 Table — (DOCX) [file pgen.1006896.s006.docx]

| si-TFAP2A | CCUGCUCACAUCACUAGUATT |
| --- | --- |
| hsa-miR-1254 mimics | AGCCUGGAAGCUGGAGCCUGCAGU |
| hsa-miR-1254 5'mt | ACGGACCUAGCUGGAGCCUGCAGU |
| hsa-miR-1254 m-mt-1 | AGCCUGGAGACUGGAGCCUGCAGU |
| hsa-miR-1254 m-mt-2 | AGCCUGGAGAUUGGAGCCUGCAGU |
| hsa-miR-1254 m-mt-3 | AGCCUGGAGAUCGGAGCCUGCAGU |
| hsa-miR-1254 m-mt-4 | AGCCUGGAGAUCAGAGCCUGCAGU |
| hsa-miR-1254 m-mt-5 | AGCCUGGAGAUCAAAGCCUGCAGU |
| hsa-miR-1254 m-mt-6 | AGCCUGGAGAUCAAGGCCUGCAGU |
| hsa-miR-1254 m-mt-7 | AGCCUGGAGAUCAAGACCUGCAGU |
| hsa-miR-1254 3'mt-1 | AGCCUGGAAGCUGGAGCCUGCGAU |
| hsa-miR-1254 3'mt-2 | AGCCUGGAAGCUGGAGCCUGUGAU |
| hsa-miR-1254 3'mt-3 | AGCCUGGAAGCUGGAGCCUAUGAU |
| hsa-miR-1254 3'mt-4 | AGCCUGGAAGCUGGAGCCCAUGAU |
| hsa-miR-1254 3'mt-5 | AGCCUGGAAGCUGGAGCUCAUGAU |
| hsa-miR-1254 mut | AGCCUGGAAGCCAAGAUUCGCAGU |
| hsa-miR-505-3p | CGUCAACACUUGCUGGUUUCCU |
| hsa-miR-22 | AAGCUGCCAGUUGAAGAACUGU |
| hsa-miR-218 | UUGUGCUUGAUCUAACCAUGU |
| hsa-miR-510 | UACUCAGGAGAGUGGCAAUCAC |
| hsa-miR-520a-5p | CUCCAGAGGGAAGUACUUUCU |
| hsa-miR-525-5p | CUCCAGAGGGAUGCACUUUCU |
| hsa-miR-373* | ACUCAAAAUGGGGGCGCUUUCC |
| hsa-miR-1291 | UGGCCCUGACUGAAGACCAGCAGU |
| hsa-miR-217 | UACUGCAUCAGGAACUGAUUGGA |
| hsa-miR-409-3p | GAAUGUUGCUCGGUGAACCCCU |
| hsa-miR-485-5p | AGAGGCUGGCCGUGAUGAAUUC |
| hsa-miR-200b | UAAUACUGCCUGGUAAUGAUGA |
| hsa-miR-200c | UAAUACUGCCGGGUAAUGAUGGA |
| hsa-miR-1304 | UUUGAGGCUACAGUGAGAUGUG |
| hsa-miR-1266 | CCUCAGGGCUGUAGAACAGGGCU |
| hsa-miR-1206 | UGUUCAUGUAGAUGUUUAAGC |
| hsa-miR-873 | GCAGGAACUUGUGAGUCUCCU |
| hsa-miR-128 | CGGGGCCGUAGCACUGUCUGAGA |
| hsa-miR-328 | GGGGGGGCAGGAGGGGCUCAGGG |
| hsa-miR-338-3p | UCCAGCAUCAGUGAUUUUGUUG |
| hsa-miR-484 | UCAGGCUCAGUCCCCUCCCGAU |
| hsa-miR-377 | AUCACACAAAGGCAACUUUUGU |
| hsa-miR-1205 | UCUGCAGGGUUUGCUUUGAG |
| hsa-miR-505* | GGGAGCCAGGAAGUAUUGAUGU |
| hsa-miR-760 | CGGCUCUGGGUCUGUGGGGA |
