# Supplementary material for: MiR-1254 suppresses HO-1 expression through seed region-dependent silencing and non-seed interaction with TFAP2A transcript to attenuate NSCLC growth
Source: PLoS Genet. 2017 Jul 27;13(7):e1006896. doi: 10.1371/journal.pgen.1006896 (PMC5549757; doi:10.1371/journal.pgen.1006896)
Supplement: S2 Table — (DOCX) [file pgen.1006896.s007.docx]

| **β-actin –realtime PCR-F** | GGCTACAGCTTCACCACCAC |
| --- | --- |
| **β-actin-realtime PCR- R** | GAGTACTTGCGCTCAGGAGG |
| **HO1-realtime PCR-F** | CCATAGGCTCCTTCCTCCTTTC |
| **HO1-realtime PCR-R** | GGCCTTCTTTCTAGAGAGGGAATT |
| **TFAP2A-realtime PCR-F** | CTCCGCCATCCCTATTAACAAG |
| **TFAP2A-realtime PCR-R** | GACCCGGAACTGAACAGAAGA |
| **USF1-realtime PCR-F** | CTGCTGTTGTTACTACCCAGG |
| **USF1-realtime PCR-R** | TCTGACTTCGGGGAATAAGGG |
| **NF-κB1-realtime PCR-F** | AACAGAGAGGATTTCGTTTCCG |
| **NF-κB1-realtime PCR-R** | TTTGACCTGAGGGTAAGACTTCT |
| **CCAR1-realtime PCR-F** | TCACAGGGGTGGTTACAAAAC |
| **CCAR1-realtime PCR-R** | CCTTTGACAGCACTAAGCTGAA |
| **U6 snRNA-realtime PCR-F** | CTCGCTTCGGCAGCACA |
| **U6 snRNA-realtime PCR-R** | AACGCTTCACGAATTTGCGT |
| **Mature miR-1254 Taqman primer** | AGCCUGGAAGCUGGAGCCUGCAGU |
| **miR-1254-CRISPR-sgRNA-left-F** | cacc g CCCAGCTACTTGGGAAGCTG |
| **miR-1254-CRISPR-sgRNA-left-R** | aaac CAGCTTCCCAAGTAGCTGGG c |
| **miR-1254-CRISPR-sgRNA-right-F** | cacc GTGTGTGTAAGGTTGCAGCT |
| **miR-1254-CRISPR-sgRNA-right-R** | aaac AGCTGCAACCTTACACACAC |
| **pri-miR-1254-1 CRISPR-PCR-F** | GCCAGGCAAGGTAGCTCATG |
| **pri-miR-1254-1 CRISPR-PCR-R** | CAAGTGATCCTCCCACCTCAGG |
| **HO-1-promoter-sgRNA-left-F** | cacc GAGCTGGAGACAGCAGAGCC |
| **HO-1-promoter-sgRNA-left-R** | aaac GGCTCTGCTGTCTCCAGCTC |
| **HO-1-promoter-sgRNA-right-F** | cacc g ATTCCAGCAGGTGACATTTT |
| **HO-1-promoter-sgRNA-right-R** | aaac AAAATGTCACCTGCTGGAAT c |
| **TFAP2A-3UTR-sgRNA-left-F** | cacc g CACACCCCTGTGCCCTCATG |
| **TFAP2A-3UTR-sgRNA-left-R** | aaac CATGAGGGCACAGGGGTGTG c |
| **TFAP2A-3UTR-sgRNA-right-F** | cacc g ACGGCCTGTTCTGTTCTCTT |
| **TFAP2A-3UTR-sgRNA-right-R** | aaac AAGAGAACAGAACAGGCCGT c |
| **HO-1-promoter-ChIP-F** | GCCAGAAAGTGGGCATCAG |
| **HO-1-promoter-ChIP-R** | CTGAGGACGCTCGAGGGAG |
| **HO-1-3UTR-luciferase-F** | ccgctcgag TTTATGCCATGTGAATGCA |
| **HO-1-3UTR-luciferase-R** | aaggaaaaaagcggccgc  AAGCTACTATCAGACAATGTTGTTT |
| **HO-1-3UTR-luciferase -mut-F** | GTAGGTCCGTATGGCCTAAACTTCATAGGGGGC |
| **HO-1-3UTR-luciferase -mut-R** | TGCTTTTCGTTGGGGAAGATGCC |
| **TFAP2A-3UTR-luciferase-F** | ctagctagc GGGTGTGAGGAGGCAAGCAGTCAGC |
| **TFAP2A-3UTR-luciferase-R** | atagtttagaggccgc CCCTGCTCTGAACTCCAAGTTG |
| **TFAP2A-3UTR- luciferase -mut -F** | caagattc AAGAGAACAGAACAGGCCGTGAAG |
| **TFAP2A-3UTR- luciferase -mut -R** | CATGAGGGCACAGGGGTGTG |
| **HO-1-promoter-luciferase -F** | ccgctcgag GCGGCCGGTCACATTTAT |
| **HO-1-promoter-luciferase -R** | ccgctcgag GCGGCCGGTCACATTTAT |
| **HO-1-promoter-luciferase-site1-F** | ctagctagc GACTGCTCCTCTCCACCCCACACTG |
| **HO-1-promoter-luciferase-site2-F** | ctagctagc TGGGACGCCTGGGGTGCATCAAGTC |
| **HO-1-promoter-luciferase-site3-F** | ctagctagc TGCCCACCAGGCTATTGCTCTGAGC |
| **HO-1-promoter-luciferase-site4-F** | ctagctagc GTGCCTGGAAGAGTGTCCCACGCAT |
| **HO-1-promoter-luciferase-site5-F** | ctagctagc GCTACCATGCCAGGCCTGAAAGCCA |
| **HO-1-promoter-luciferase-site6-F** | ctagctagc GAGGGACAGCGTCTTGTTCTGTTGCC |
| **HO-1-promoter-luciferase-mt1-1-F** | CCGCTCGAGGATATCAAGATCTGGCCTCGG |
| **HO-1-promoter-luciferase-mt1-1-R** | GGTCACATTTATGCTCGGCGGGTCACGTGG |
| **HO-1-promoter-luciferase-mt1-2-F** | GTTCCGCCTGGCCCACGTGACCC |
| **HO-1-promoter-luciferase-mt1-2-R** | CACTTTCTGGCCGGGCGTTGCAACACC |
| **TFAP2A-CDS-PTT5-F** | ctagctagc ATGTTAGTTCACAGTTTTTCAGCCATGGAC |
| **TFAP2A-CDS-PTT5-R** | atagtttagcggccgc  TCACTTTCTGTGCTTCTCCTCTTTGTCA |
| **HO1-CDS-PTT5-F** | ccggaattc ATGGAGCGTCCGCAACCC |
| **HO1-CDS-PTT5-R** | cccaagctt TCACATGGCATAAAGCCCTACAGCA |
